# The Optimal Chinese Herbal Injections for Use With Radiotherapy to Treat Esophageal Cancer: A Systematic Review and Bayesian Network Meta-Analysis

**DOI:** 10.3389/fphar.2018.01470

**Published:** 2019-01-04

**Authors:** Dan Zhang, Mengwei Ni, Jiarui Wu, Shuyu Liu, Ziqi Meng, Jinhui Tian, Xiaomeng Zhang, Bing Zhang

**Affiliations:** ^1^Department of Clinical Chinese Pharmacy, School of Chinese Materia Medica, Beijing University of Chinese Medicine, Beijing, China; ^2^Evidence Based Medicine Center, Lanzhou University, Lanzhou, China

**Keywords:** Chinese herbal injections, radiotherapy, esophageal cancer, systematic review, network meta-analysis

## Abstract

**Ethnopharmacological relevance:** Esophageal cancer is one of the most common human cancers, and its incidence is highly endemic in China. The combination of Chinese herbal injections (CHIs) and radiotherapy should be informed by the best available evidence.

**Aim of the study:** To update and expand on previous work in order to compare and rank the efficacy and safety of CHIs in combination with radiotherapy to treat esophageal cancer.

**Materials and Methods:** We searched several electronic databases to identify randomized controlled trials (RCTs) regarding CHIs to treat esophageal cancer from their inception to March 15, 2017. In a network meta-analysis (NMA), the bias of the included trials was assessed by two individuals independently in accordance with the Cochrane Handbook for Systematic Reviews of Interventions. Outcomes such as the clinical effectiveness rate, performance status, adverse reactions (ADRs), and survival rate were evaluated. We performed a random-effects NMA to obtain estimates of efficacy and safety outcomes, and we present these estimates as odds ratios (ORs) and corresponding 95% confidence intervals (CIs) calculated via Stata 13.1 and WinBUGS 1.4 software. Furthermore, the surface under cumulative the ranking curve (SUCRA) was used to rank the efficacy and safety of different CHIs in relation to each outcome.

**Results:** Of 685 identified trials, 55 were eligible for inclusion in the study. These 55 trials included 12 CHIs and 4,114 participants. The cluster analysis results suggested that Compound kushen injection therapy is the optimal CHI treatment for patients with esophageal cancer in terms of improving the clinical effectiveness rate and performance status. Huachansu and Kangai injection are superior in improving 1-year and 2-year survival rates. Lentinan injection may be considered a favorable choice for reliving ADRs, and Compound kushen injection may provide treatment benefits by reducing both gastrointestinal reaction and radiation esophagitis.

**Conclusions:** The current clinical evidence indicated that Compound kushen injection combined with radiotherapy is the most preferable and beneficial option for patients with esophageal cancer in terms of efficacy and safety. However, the results of our study should be interpreted with caution given the limitations of the sample size and the methodological quality of the included trials.

## Introduction

Esophageal cancer, the eighth most common cancer worldwide, is extremely aggressive and has a low survival rate (Pennathur et al., 2013; Zacherl, 2014). Esophageal cancer is the sixth leading cause of death among cancers, with an estimated 400,200 deaths, and ~49.35% of cases occur in China (197,500 deaths; Ferlay et al., 2015). Since the 1990s, esophageal cancer has ranked fourth in both the number of cancer diagnoses and cancer mortalities in China; thus, it represents a heavy disease burden (Wang et al., 2012; Lin et al., 2013). Although surgery remains the primary treatment for localized esophageal cancer, the majority of patients recur in regional or distant sites after radical resection, and the overall 5-year survival rate ranges from 15 to 25% (Cappetta et al., 2012; Zhang et al., 2016). An increasing number of patients are diagnosed with esophageal cancer at the advanced stages, and some cases can benefit from radiotherapy to improve overall and progression-free survival as well as locoregional control (Shridhar et al., 2013; Nguyen et al., 2014). However, studies have demonstrated that radiotherapy is often accompanied by serious adverse reaction (ADRs), further impairing the health-related performance status of cancer patients (Beukema et al., 2015). To address this relevant issue, traditional Chinese Medicine (TCM) as an adjuvant cancer treatment has exhibited increased efficacy and reduced side effects (Xu et al., 2015; Cai et al., 2017). In recent decades, inducing cancer apoptosis with TCM has become increasingly popular in the field of oncology (Zhang et al., 2016).

With the transformation of Chinese herbal treatment from traditional preparation to modern preparation, Chinese herbal injections (CHIs) are poised to be a considerable adjuvant treatment for esophageal cancer in combination with radiotherapy or chemotherapy; they have been shown to improve clinical symptoms and nutritional status, inhibit cancer progression, and offer manageable safety profiles (Chung et al., 2015; Wu et al., 2016; Zhang et al., 2017b). Numerous biological and pharmacological studies have been conducted to explain the mechanism of CHIs against cancer. For example, Aidi injection during radiotherapy can effectively inhibit the Th1/Th2 shift among patients with esophageal cancer (Wang and Chen, 2009). However, the types of CHI are various, and the optimal strategy for combining CHIs with radiotherapy to treat esophageal cancer remains inconclusive. Network meta-analysis (NMA) can integrate direct and indirect comparisons based on clinical trials and simultaneously pool evidence on various interventions to rank their comparative efficacy and safety (Salanti et al., 2014). Recently, NMA has been accepted as a key part of the health care decision-making process due to its superiority in drug evaluation (Laws et al., 2014). Given that CHIs combined with radiotherapy are available for esophageal cancer patients, and the absence of clinical trials that directly compare the different types of CHIs, an NMA was designed and implemented in the present study to fill this knowledge gap. The NMA compared the efficacy and safety of combining CHIs with radiotherapy to treat esophageal cancer by quantitatively synthesizing the evidence. The aim was to identify the optimal strategy for treating esophageal cancer and to strengthen inferences for clinical practice in future.

## Materials and Methods

The procedure of the current NMA was performed in agreement with the Preferred Reporting Items for Systematic Reviews and Meta-Analyses (PRISMA) guidelines (Liberati et al., 2009).

### Database and Retrieval Strategies

The consulted electronic databases included Embase, PubMed, the Cochrane Library, the China National Knowledge Infrastructure Database (CNKI), the Wan-fang Database, the Cqvip Database (VIP), and the China Biology Medicine disc (CBMdisc). The retrieval period was from inception to March 15, 2017. No limitations were placed on the publication year, language, or blinding methods. To identify relevant publications, search terms were constructed for three domains: (1) esophageal cancer, (2) CHIs, and (3) study type (randomized controlled trials). The following terms were used for esophageal cancer: “Esophageal Neoplasms [MeSH Terms],” “Esophageal Neoplasm,” “Esophagus Neoplasm,” “Esophagus Neoplasms,” “Cancer of Esophagus,” “Cancer of the Esophagus,” “Esophagus Cancer,” “Esophagus Cancers,” “Esophageal Cancer,” and “Esophageal Cancers.” More specific retrieval strategies are provided in the supplementary file, Table S1. The retrieval strategies were appropriately developed and adopted in consultation with a reference librarian at our university. The reference lists of identified systematic reviews and meta-analyses were further searched to locate potential RCTs (Tables S2, S3).

### Inclusion and Exclusion Criteria

The PICOS (patients, intervention, comparison, outcome, and study design) framework was used to select potentially relevant studies. All articles were independently reviewed by two investigators (DZ, MN). RCTs were included if they satisfied the following criteria: (1) The study included patients affected by esophageal cancer, without limitations on gender, age, race, region, or nationality. (2) The interventions involved the administration of CHIs combined with radiotherapy in either arm of treatment, and the CHIs used (such as Aidi injection, Compound kushen injection, and Kangai injection) were authorized by the China Food and Drug Administration and applied in clinics for treating tumors. (3) The study performed a comparison with esophageal cancer patients receiving only radiotherapy, regardless of its course or dosage. (4) The study described efficacy outcomes, such as the clinical effectiveness rate, performance status, and 1-year and 2-year survival rate, and the safety outcomes were ADRs, such as leucopenia, gastrointestinal reactions, and radiation esophagitis. (5) The study was a RCT that compared the relative outcomes of CHIs combined with radiotherapy.

Two investigators (JW, SL) independently perused the titles and abstracts of the identified RCTs and excluded irrelevant clinical trials. The exclusion criteria were as follows: (1) Patients were with other tumors. (2) The interventions included surgery, chemotherapy or other cancer treatments; CHIs were not combined with radiotherapy in either arm; arms from the same trials were different in their therapy duration or drug administration. (3) Relevant outcome indexes were not reported or estimated. (4) The study designs and publication types were non-RCT (for example, qualitative studies, observational studies, meta-analyses, case reports, single-arm trials, pharmacological experiments, or reviews) or were duplicates, and or the full text was unavailable.

### Data Extraction and Quality Assessment

All potential articles were managed and organized via EndNote X7 software (Thomson Reuters Crp.3 Times Square, New York, The United States). After removing duplicate records, two investigators (DZ, MN) independently screened the initial search results for potentially eligible studies. All identified articles were then retrieved in full, and the following data were extracted using Microsoft Excel (Microsoft Corp, Redmond, WA): (1) Publication information: the name of the first author and the publication year. (2) Characteristics of the enrolled patients with esophageal cancer: number, age, gender, and the type and stage of cancer. (3) Information on the intervention: dosage, duration, and treatment cycle. (4) Outcomes: the measured data on the efficacy and safety outcomes. These outcomes were calculated using the following formula: the clinical effectiveness rate = (number of complete response patients+number of partial response patients)/total number of patients × 100%) (World Health Organization, 1979). Performance status was evaluated by the Karnofsky performance score (KPS). An improvement in performance status was defined as a KPSs increase of more than 10 points. Survival rate = (number of surviving patients who were followed up with for 1 or 2 years/number of total patients) × 100%. With regard to ADRs, the incidence of ADRs = (number of patients with ADRs/total number of patients) × 100%. (5) Description of study design: blinding, randomized allocation methods, and other items for quality assessment.

Risk-of-bias assessments were completed by two investigators (JW and SL) independently for all individual studies using the Cochrane Risk of Bias Tool [Cochrane Handbook for Systematic Reviews of Interventions, version 5.1.0; (Higgins et al., 2011)] through Review Manager (RevMan) version 5.3 statistical software (The Cochrane Collaboration 2014, Nordic Cochrane Centre Copenhagen, Denmark). Discrepancies were resolved either by consensus or through adjudication by a third investigator (DZ). The quality evaluation of the included RCTs focused on several key domains: selection bias (random sequence generation and allocation concealment), performance bias (blinding of participants and personnel), detection bias (blinding of outcome assessment), attrition bias (incomplete outcome data), reporting bias (selective reporting) and other bias. Each of these items was assessed as high, low or unclear. Information on randomized methods, follow-up, blind methods, allocation concealment, reasons for withdrawal, inclusion and exclusion criteria, ADRs, statistical methods, foundations, and medical ethics for each RCT was also used for the quality evaluation.

The present NMA did not require ethical approval because it gathered data from previously published trials.

### Statistical Analysis

We expressed the comparative efficacy and safety of the treatments as the relative risk for dichotomous outcomes with 95% confidence intervals (CIs). The differences between the compared groups were significant, as the 95% CIs did not contain 1. A Bayesian NMA was designed to effectively increase the sample size and provide valid pooled effect estimates for different types of CHIs combined with radiotherapy to treat esophageal cancer. WinBUGS 1.4.3 software (MRC Biostatistics Unit, Cambridge, UK) was utilized to perform a statistical analysis. The Bayesian approach provided probabilistic distributions of the estimates of interest through a large number of simulations and hence produced results with intuitive interpretations through the Markov Chain Monto Carlo method in the random-effects model (Achana et al., 2014; Stephenson et al., 2015; Greco et al., 2016). The choice of the random-effects model for outcomes was mainly driven by the within-study and between-study methodological and clinical variation in the current NMA (Jackson et al., 2014; Chan, 2016). The results of analysis procedure were based on 200,000 simulation iterations and 10,000 adaptation iterations. Additionally, Stata version 13.1 software (Stata Corp, College Station, TX) was adopted to present the results and graphs of the NMA (Shim et al., 2017). The network graph displayed the relationships in the observed comparisons. The thickness of the lines in the network graph was proportional to the number of trials used for the comparisons, and the node sizes corresponded to the total sample sizes for the treatments (Chaimani et al., 2013; Donegan et al., 2013). Moreover, the surface under the cumulative ranking (SUCRA) curve was employed to rank the different CHIs in relation to each outcome. The SUCRA value ranged from 0 to 100%, with a larger SUCRA value indicating a better treatment option (Rücker and Schwarzer, 2015; Trinquart et al., 2016). Publication bias was graphically assessed via a comparison-adjusted funnel plot (Trinquart et al., 2012; Krahn et al., 2014). In addition, if there were closed loops among the included RCTs, the inconsistency between indirect and direct comparisons was calculated with the inconsistency factors and their 95% CIs in a node-splitting analysis for each loop of evidence (Mavridis et al., 2014; Piepho, 2014). A cluster analysis was also performed to synthesize the efficacy and safety of each treatment (reporting two different outcomes) simultaneously. Interventions located in the upper-right corner were superior to others (Veroniki et al., 2015).

## Results

### Literature Search and Study Characteristics

**Figure 1:**
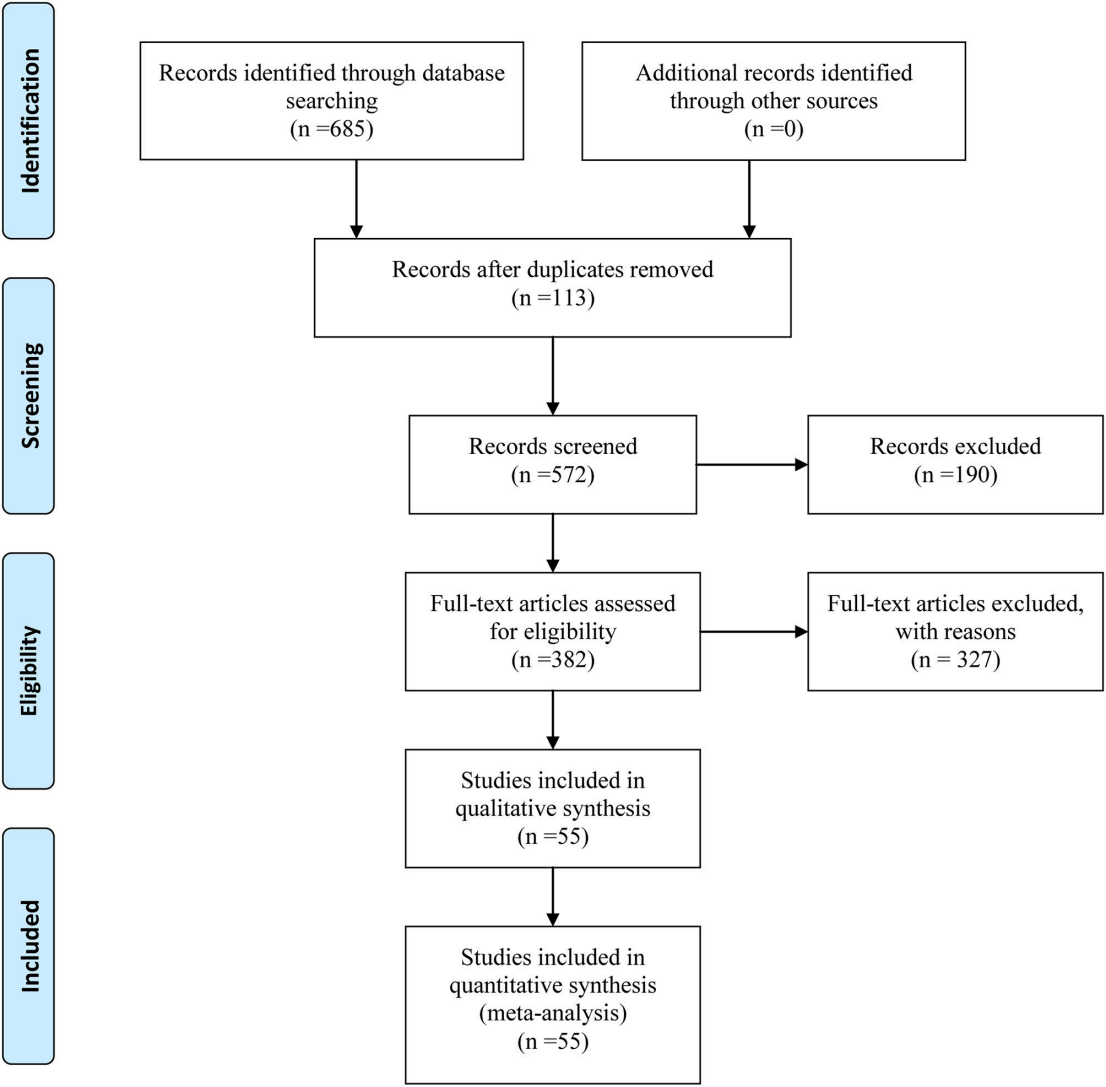
Flow chart of the search for eligible studies.

Initially, our above-described search strategy yielded 685 citations from electronic databases. After duplicates and irrelevant articles were removed, 282 studies remained, and through further inspection, a total of 55 RCTs involving 12 CHIs met our selection criteria. These RCTs were included in the current NMA, and their details are provided in File S1. The study identification, screening, and inclusion process is illustrated in Figure 1. The number of studies included for different CHIs was as follows: Aidi injection (15 trials), compound kushen injection (11 trials), Javanica oil emulsion injection (10 trials), Kangai injection (7 trials), Huachansu injection (4 trials), Elemene injection (2 trials), Astragalus polysaccharide injection (1 trial), Astragalus injection (1 trial), Lentinan injection (1 trial), Shenmai injection (1 trial), Disodium cantharidinate and vitamin B6 injection (1 trial), and Shenqifuzheng injection (1 trial).

**Figure 2:**
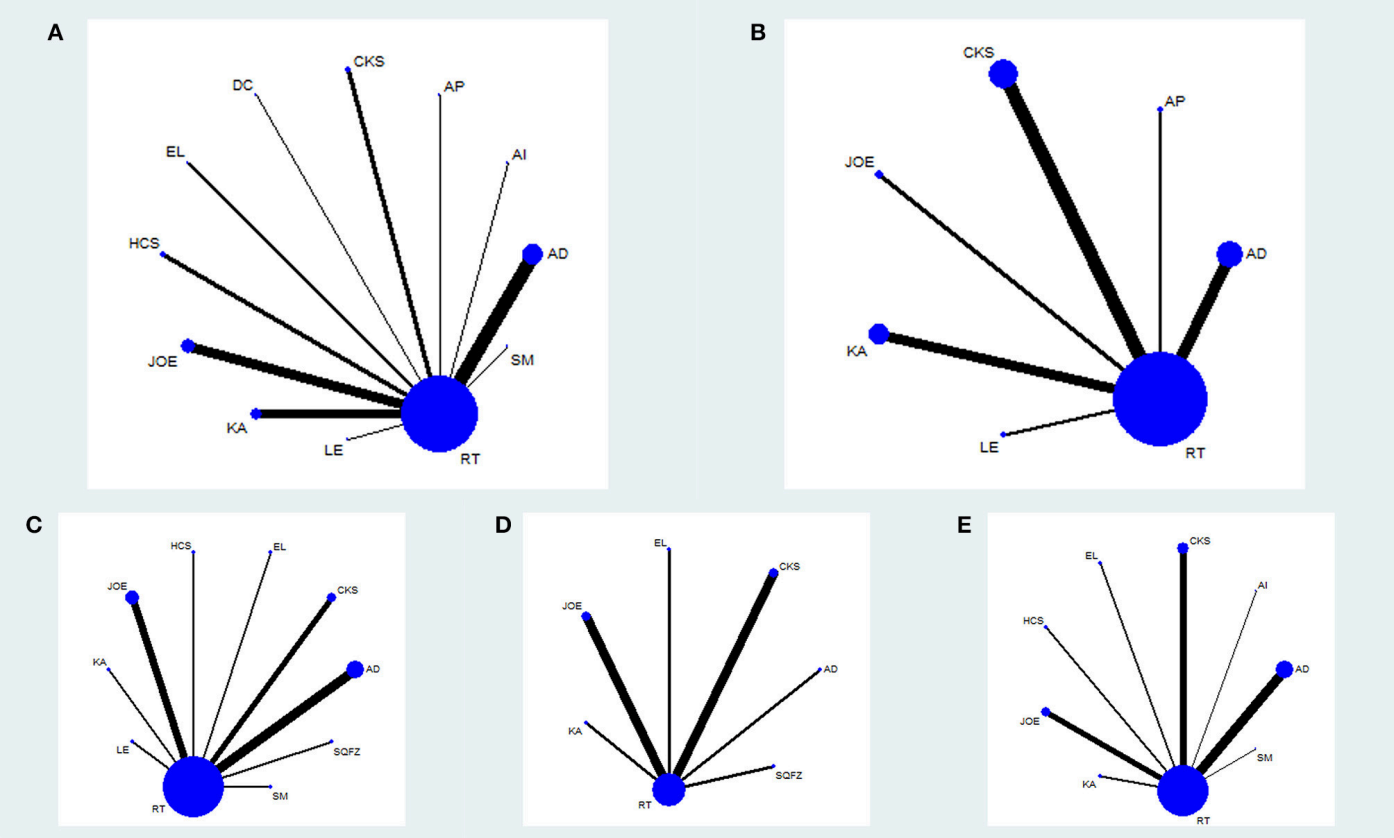
Network graph for different outcomes. Node sizes indicate total sample sizes for treatments. Line thicknesses correspond to the number of trials used for comparisons. **(A)** clinical effectiveness rate; **(B)** performance status; **(C)** leucopenia; **(D)** gastrointestinal reactions; **(E)** radiation esophagitis.

The baseline characteristics of each RCT included were summarized in Table 1. Overall, the 55 trials involved 4,114 patients with esophageal cancer; 2,073 of them received a combination of CHIs and radiotherapy in an experimental group, and 2,041 patients received only radiotherapy in a control group. All RCTs reported the sample size and the age of patients, and 49 trials (89.09%), 15 trials (27.27%), 17 trials (30.91%), and 39 trials (70.91%) provided information on gender, TNM stages, expected survival time, and KPS score before treatment, respectively. Figure 2 depicted the network plot of the interventions included in the Bayesian analysis.

**Table 1:** Basic Characteristics of the Included Studies.

**Study ID**	**Sex (M/F)**	**AVG age**	**N (E/C)**	**TNM stages**	**EST (m)**	**KPS score**	**Therapy of CHIs+radiotherapy group**	**Therapy of radiotherapy group**	**Treatment (days)**	**Outcomes**
Yang YM 2013	38/22	48	30/30	I–III	≥3	>60	AD 100 ml+ DT = 66Gy	DT = 66Gy	30d	① ②
Fang H 2011	38/22	48	30/30	I–III	≥3	>60	AD 100 ml+ DT = 66Gy	DT = 66Gy	30d	① ②
Zeng QB 2006	NR	NR	72/72	NR	NR	NR	AD 100 ml+ DT = 60–70Gy	DT = 60–70Gy	15d × (2–3)	① ④
Mao HY 2016	80/30	65	55/55	NR	≥3	≥60	AD 100 ml+ DT = 50–66Gy	DT = 50–66Gy	30d	① ③
Zhao KY 2010	54/16	57	40/30	II–IV	≥5	>60	AD 50 ml+ DT = 60–70Gy	DT = 60–70Gy	7d × (6–7)	① ②
Guo YC 2014	56/40	57 ± 5	50/46	II–IV	≥6	≥70	AD 50 ml+ DT = 60–66Gy	DT = 60–66Gy	7d × (6–7)	① ③
Li MJ 2014	43/17	58	30/30	II–IV	>6	≥60	AD 50 ml+ DT = 60Gy	DT = 60Gy	5d × 4	① ③
Lu K 2006	56/29	56	43/42	II–III	NR	≥70	AD 60 ml+ DT = 60–70Gy	DT = 60–70Gy	21d	① ③
Hu LM 2010	42/26	58.4	34/34	NR	NR	≥70	AD 50 ml+ DT = 64–70Gy	DT = 64–70Gy	7d × (6–7)	③ ④
Bai LK 2014	33/16	75.4	25/24	NR	NR	>60	AD 50 ml+ DT = 50–70 Gy	DT = 50–70 Gy	7d × (4–7)	③ ④
Zhao X 2015	40/32	NR	36/36	NR	≥6	≥70	AD 50 ml+ DT = 64Gy	DT = 64Gy	7d × (6–7)	③ ④
Han JW 2008	NR	71.5	48/47	II–IV	NR	≥70	AD 50 ml+ DT = 65–70Gy	DT = 65–70Gy	7d × (6–7)	① ③
Xing HJ 2011	46/34	70.5	40/40	NR	>3	≥70	AD50 ml+ DT = 60–70Gy	DT = 60–70Gy	7d × (6–7)	① ② ③ ④
Wu YH 2001	NR	NR	48/48	NR	NR	>60	AD 30–50 ml+ DT = 68–70Gy	DT = 68–70Gy	7d × (6–7)	① ③ ④
Liu XP 2006	36/12	75	24/24	NR	NR	≥60	AD 60 ml+ DT = 60–70Gy	DT = 60–70Gy	7d × (6–7)	① ② ③
Jiang SN 2010	36/14	64.5	26/24	NR	NR	70–90	DC 0.5–1.0 mg+ DT = 60Gy	DT = 60Gy	7d × 6	①
Zhou M 2009	44/19	51.5	32/31	NR	NR	≥70	SM 50 ml+ DT = 60Gy	DT = 60Gy	10d × 3	① ③ ④
Zhu GJ 2013	NR	NR	30/30	NR	NR	60–80	SQFZ 250 ml+ DT = 64Gy	DT = 64Gy	28d	③ ④
Cai P 2006	98/28	NR	53/73	NR	NR	>60	CKS 20 ml+ DT = 66–74Gy	DT = 66–74Gy	20d	③ ④
Yan L 2015	45/35	55	40/40	I–IV	NR	≥70	CKS 20 ml+ DT = 60–66Gy	DT = 60–66Gy	14d	② ③
Sun TZ 2009	53/17	55.5	35/35	NR	NR	>60	CKS 20 ml+ DT = 60–70Gy	DT = 60–70Gy	21d	③ ④
Huang CH2016	49/31	70–80	40/40	NR	NR	≥70	CKS 15–20 ml+DT = 60–66Gy	DT = 60–66Gy	7d × (6–7)	③ ④
Ao JF 2006	21/11	56.8	18/14	III–IV	NR	≥60	CKS 20 ml+ DT = 60–70Gy	DT = 60–70Gy	7d × (4–6)	① ③
Chen XY 2006	54/13	55	33/35	NR	NR	≥60	CKS20–40 ml+DT = 66.6–67Gy	DT = 66.6–67Gy	10d × (3–4)	① ② ③ ④
Sheng ZJ 2009	67/61	72.2	66/62	III	NR	NR	CKS 20 ml+ DT = 60Gy	DT = 60Gy	(14–35)d	② ③
Ding JQ 2011	23/25	55.2	24/24	NR	NR	≥70	CKS 20 ml+ DT = 65.4–67Gy	DT = 65.4–67Gy	20d	② ③ ④
Li Z 2012	48/19	63	35/32	II–III	>3	>60	CKS 15 ml+ DT = 60–66Gy	DT = 60–66Gy	14d × (2–3)	① ② ③
Luo M 2013	NR	NR	26/23	NR	>3	>70	CKS 25 ml+ DT = 50–66Gy	DT = 50–66Gy	7d × (5–7)	③
Liu FX 2009	66/34	53.2	50/50	NR	NR	≥60	CKS 20 ml+ DT = 54–66Gy	DT = 54–66Gy	(14–21)d	②
Zhou DA 2002	116/44	61.9	80/80	NR	NR	≥70	HCS 20 mL+ DT = 60–70Gy	DT = 60–70Gy	28d	①
Tian SP 2013	12/10	72–89	10/12	NR	>6	>70	CHS 20 mL+ DT = 60Gy	DT = 60Gy	7d × 6	③
Zhang FT 2001	32/28	55	30/30	NR	>3	>70	CHS 40 mL+ DT = 60–70Gy	DT = 60–70Gy	7d × (6–7)	① ④
Wang ZM 2010	34/22	68	28/28	NR	NR	≥70	CHS20–30 mL+DT = 40–50Gy	DT = 40–50Gy	(21–28)d	① ② ③
He WX 2007	38/22	48	30/30	I–III	≥3	>60	AP 250 ml+ DT = 66Gy	DT = 66Gy	2ld	① ②
Fan T 2012	NR	NR	60/60	NR	NR	≥60	AI 30 ml+ DT = 60–70Gy	DT = 60–70Gy	7d × (6–7)	① ③
An SH 2008	35/5	71	20/20	NR	NR	NR	KA 40 ml+ DT = 60–64Gy	DT = 60–64Gy	7d × (6–7)	②
Zhao DL 2006	38/22	48	30/30	I–III	NR	NR	KA 60 ml+ DT = 66Gy	DT = 66Gy	7d × (6–7)	① ②
Wang WH2014	49/29	55.9	39/39	III–IV	≥3	≥70	KA 60 ml+ DT = 50–70Gy	DT = 50–70Gy	28d × 2	① ③
Mu Y 2012	32/18	54	25/25	NR	NR	≥70	KA 60 ml+ DT = 60–70Gy	DT = 60–70Gy	7d × 6	① ②
Zhang HF 2014	39/11	61	25/25	NR	NR	NR	KA 50 ml+ DT = 60–66Gy	DT = 60–66Gy	7d × (6–7)	① ③
Wu ZP 2013	56/31	72.3	43/44	NR	>3	>60	KA 60 ml+ DT = 60–70Gy	DT = 60–70Gy	7d × (6–7)	① ② ③ ④
Ren MZ 2013	39/15	NR	28/26	NR	NR	≥60	KA 40–60 ml+ DT = 60Gy	DT = 60Gy	30d × 2	① ③
Ding H2013	59/21	58.5	40/40	NR	NR	≥80	EL 200 mg+ DT = 50–60Gy	DT = 50–60Gy	7d × (6–7)	① ③
Zhu XG 2016	51/41	67.6	50/42	II–III	NR	NR	EL 600 mg+ DT = 50–60Gy	DT = 50–60Gy	30d × 2	① ③ ④
Wu J 2011	37/23	56	30/30	NR	>3	>70	LE lmg+ DT≥ 45Gy	DT≥45Gy	7d × 6	① ② ③
Feng SJ 2015	35/25	65.5	30/30	NR	NR	NR	JOE 30 ml+ DT = 60Gy	DT = 60Gy	21d × 3	① ③
Li DZ 2011	37/19	53	28/28	NR	NR	≥70	JOE 30 ml+ DT = 50–60Gy	DT = 50–60Gy	21d	① ④
Li Q 2013	36/14	62	25/25	NR	>12	≥70	JOE 30 ml+ DT = 60Gy	DT = 60Gy	7d × 6	① ④
Jia YS 2008	116/32	55.6	76/72	NR	NR	≥70	JOE 30–50 ml+ DT = 60–68Gy	DT = 60–68Gy	30d	② ③ ④
Chen SD 2007	65/27	NR	44/48	NR	NR	≥70	JOE 10–20 ml+ DT = 64–70Gy	DT = 64–70Gy	7d × (6–7)	③
Jiang XC 2009	41/28	55.6	35/34	NR	NR	≥70	JOE 30 ml+ DT = 60–70Gy	DT = 60–70Gy	7d × (6–7)	① ③
Kong XM 2004	47/13	55.6	30/30	NR	NR	>60	JOE 30 ml+ DT = 60–70Gy	DT = 60–70Gy	21d × 2	① ③ ④
Liu XX 2010	39/17	61	28/28	NR	NR	60–80	JOE 30 ml+ DT = 60–70Gy	DT = 60–70Gy	21d × 2	① ③
He LJ 2010	63/7	63	35/35	NR	NR	≥60	JOE 30 ml + DT = 60–70Gy	DT = 60–70Gy	7d × (6–7)	① ③
Qi JH 2015	68/42	61.8	61/49	NR	NR	≥60	JOE 20–30 ml+ DT = 60–66Gy	DT = 60–66Gy	7d × (6–7)	① ②

*M, male; F, female; E, experimental group; C, control group; NR, not reported; EST, expected survival time; m, month; d, day; c, cycle; ①, clinical effectiveness rate; ②, performance status, ③, ADRs; ④, survival rate; AD, Aidi injection; AI, Astragalus injection; AP, Astragalus polysaccharide injection; CKS, Compound kushen injection; DC, Disodium cantharidinate and vitamin B6 injection; EL, Elemene injection; HCS, Huachansu injection; JOE, Javanica oil emulsion injection; KA, Kangai injection; LE, Lentinan injection; SM, Shenmai injection; SQFZ, Shenqifuzheng injection; DT, radiation absorbed dose*.

### Quality Assessment

**Figure 3:**
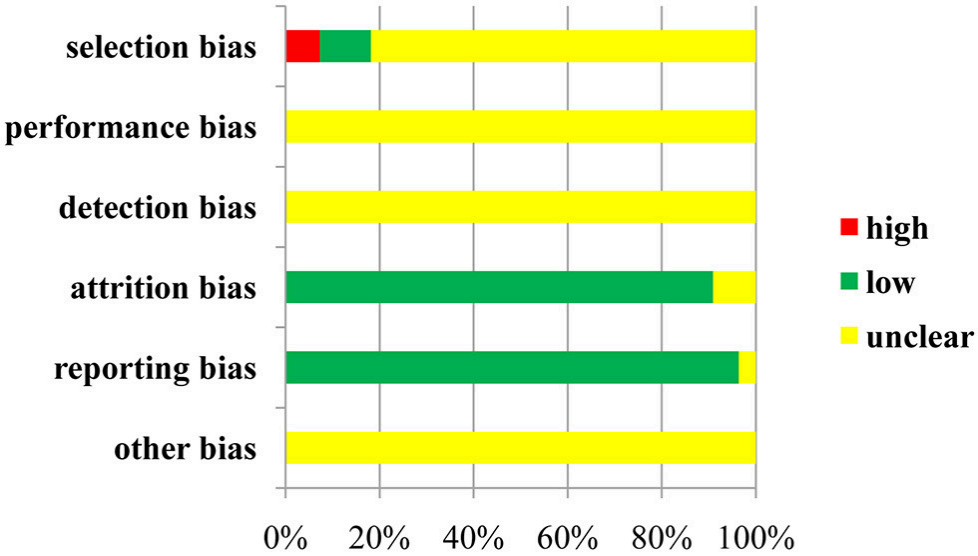
Risk-of-bias graph.

Regarding selection bias, 6 (10.91%) of 55 trials were rated as low risk because they adopted random number tables and stratified blocks, and 4 trials (7.27%) were regarded as high risk because they used the method of hospital time difference. The risk of remaining RCTs was deemed unclear. Not all of the included trials described the allocation concealment and blinding method, and not all of them provided information on the attrition bias and reporting bias.With respect to other bias, the included trials did not provide the messages; therefore, they were identified as unclear in this domain. A risk-of-bias graph is presented in Figure 3, and a risk-of-bias summary is shown in Figure S1.

In addition, the results of quality evaluation for the included RCTs indicated that 10 RCTs (18.18%) reported the details on the randomized method, and 17 trials (30.91%), 47 trials (85.45%), 39 trials (70.91%), 1 trial (1.82%), and 9 trials (16.36%) provided information on follow-up, ADRs, statistical methods, foundations and medical ethics, respectively. All RCTs identified the inclusion and exclusion criteria for patients with esophageal cancer, whereas none of them described the impact of the blind method, allocation concealment, and reasons for withdrawal. In general terms, the overall quality of the studies included in this review can be considered moderate (File S2).

### Outcomes

#### Efficacy Outcomes

**Figure 4:**
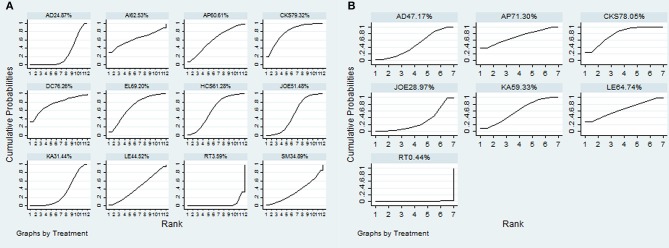
SUCRA for the treatments of clinical effectiveness rate **(A)** and performance status **(B)**.

A total of 38 RCTs with 11 types of CHIs contributed to the evidence network for the efficacy outcomes. The results suggested that 7 types of CHIs—Aidi, Disodium cantharidinate and vitamin B6, Compound kushen, Huachansu, Astragalus polysaccharide, Kangai, and Elemene injection—in combination with radiotherapy exhibited higher clinical effectiveness rates than radiotherapy alone. In addition, significant differences were detected across these 7 types of CHIs compared with radiotherapy, with ORs and 95% CIs of 0.57 (0.42, 0.78), 5.84 (1.02, 48.01), 5.21 (1.97, 15.43), 3.24 (1.83, 5.90), 3.36 (1.10, 11.84), 1.94 (1.20, 3.20), 4.00 (1.53, 11.18), respectively (Table 2). Figure 4A presents the cumulative probabilities (SUCRA values) that 11 types of CHIs combined with radiotherapy are the best option for improving the clinical effectiveness rate. The combination of Compound kushen injection and radiotherapy had the highest probability of being the best option for improving the clinical effectiveness rate (79.32%), followed by Disodium cantharidinate and vitamin B6 injection plus radiotherapy (76.26%) and Elemene injection plus radiotherapy (69.2%). The SUCRA values of each intervention in terms of different outcomes were summarized in Table 3.

**Table 2:** Results of the network meta-analysis of the clinical effectiveness rate (upper-right quadrant) and performance status (lower-left quadrant).

**AD+RT**	**0.57** **(0.42, 0.78)**	3.36 (0.56, 28.18)	1.09 (0.28, 4.38)	**3.01** **(1.07, 9.15)**	1.85 (0.96, 3.65)	1.93 (0.60, 7.05)	2.43 (0.23, 69.2)	1.11 (0.63, 1.98)	2.28 (0.83, 6.71)	1.37 (0.43, 4.55)	1.55 (0.92, 2.56)
**0.32 (0.18, 0.56)**	**RT**	**5.84** **(1.02, 48.01)**	1.91 (0.52, 7.45)	**5.21** **(1.97, 15.43)**	**3.24** **(1.83, 5.90)**	**3.36** **(1.10, 11.84)**	4.25 (0.39, 118.3)	**1.94** **(1.20, 3.20)**	**4.00** **(1.53, 11.18)**	2.41 (0.78, 7.57)	2.71 (1.82, 4.05)
–	–	**DC+RT**	0.32 (0.027, 3.07)	0.90 (0.092, 6.67)	0.55 (0.064, 3.57)	0.58 (0.049, 4.79)	0.72 (0.030, 30.41)	0.32 (0.039, 2.05)	0.69 (0.070, 5.06)	0.41 (0.038, 3.36)	0.46 (0.055, 2.81)
–	–	–	**SM+RT**	2.79 (0.49, 14.54)	1.69 (0.39, 7.20)	1.80 (0.31, 10.63)	2.25 (0.15, 66.69)	1.01 (0.24, 4.07)	2.09 (0.40, 11.02)	1.27 (0.21, 7.09)	1.41 (0.34, 5.71)
1.54 (0.75, 3.18)	**4.84** **(3.08, 7.77)**	–	–	**CKS+RT**	0.61 (0.18, 1.99)	0.64 (0.14, 3.05)	0.83 (0.058, 26.34)	0.37 (0.12, 1.10)	0.76 (0.18, 3.09)	0.46 (0.094, 2.04)	0.52 (0.16, 1.48)
–	–	–	–	–	**HCS+RT**	1.04 (0.29, 4.18)	1.32 (0.12, 39.91)	0.60 (0.28, 1.30)	1.23 (0.40, 4.05)	0.74 (0.20, 2.66)	0.84 (0.41, 1.71)
1.55 (0.39, 6.37)	**4.84** **(1.40, 18.34)**	–	–	0.99 (0.27, 4.06)	–	**AP+RT**	1.25 (0.090, 39.41)	0.58 (0.15, 1.93)	1.18 (0.25, 5.36)	0.71 (0.14, 3.50)	0.81 (0.21, 2.62)
–	–	–	–	–	–	–	**AI+RT**	0.45 (0.016, 5.21)	0.93 (0.032, 11.65)	0.56 (0.018, 7.99)	0.63 (0.022, 7.28)
1.19 (0.51, 2.82)	**3.75** **(1.98, 7.12)**	–	–	0.77 (0.35, 1.70)	–	0.77 (0.18, 3.12)	–	**KA+RT**	2.08 (0.70, 6.34)	1.25 (0.36, 4.34)	1.39 (0.75, 2.65)
–	–	–	–	–	–	–	–	–	**EL+RT**	0.60 (0.13, 2.60)	0.67 (0.23, 1.92)
1.34 (0.34, 5.68)	**4.22** **(1.21, 15.97)**	–	–	0.87 (0.23, 3.56)	–	0.87 (0.14, 5.34)	–	1.12 (0.27, 4.95)	–	**LE+RT**	1.13 (0.33, 3.67)
0.74 (0.32, 1.72)	**2.33** **(1.25, 4.37)**	–	–	0.48 (0.22, 1.03)	–	0.48 (0.11, 1.95)	–	0.62 (0.26, 1.51)	–	0.55 (0.13, 2.18)	**JOE+RT**


**Table 3:** SUCRA values of different interventions for outcomes.

	**Clinical effectiveness rate**	**Performance status**	**Leucopenia**	**Gastrointestinal reactions**	**Radiation esophagitis**	**1-year survival rate**	**2-year survival rate**
AD+RT	24.87%	47.17%	44.15%	36.56%	42.9%	52.8%	NR
RT	3.59%	0.44%	10.75%	24.23%	10.00%	3.51%	12.65%
AI+RT	62.53%	NR	NR	NR	70.81%	NR	NR
AP+RT	60.61%	71.3%	NR	NR	NR	NR	NR
CKS+RT	79.32%	78.05%	64.84%	65.84%	91.44%	38.04%	50.06%
DC+RT	76.26%	NR	NR	NR	NR	NR	NR
EL+RT	69.2%	NR	10.93%	20.96%	18.19%	32.83%	30.32%
HCS+RT	61.28%	NR	31.47%	NR	57.83%	75.22%	79.08%
JOE+RT	51.48%	28.97%	62.08%	60.53%	58.31%	52.83%	49.9%
KA+RT	31.44%	59.33%	73.28%	53.61%	55.86%	70.63%	77.98%
LE+RT	44.52%	64.74%	84.72%	NR	NR	NR	NR
SM+RT	34.89%	NR	39.95%	NR	44.65%	38.63%	NR
SQFZ+RT	NR	NR	77.83%	88.26%	NR	85.52%	NR

A total of 19 RCTs reported information on performance status across the 6 types of CHIs. The results demonstrated that patients receiving 6 types of CHIs—Aidi, Compound kushen, Astragalus polysaccharide, Kangai, Lentinan, and Javanica oil emulsion injection—plus radiotherapy presented considerable improvements in performance status relative to those with radiotherapy alone. There were significant differences between these groups, with ORs and 95% CIs of 0.32 (0.18, 0.56), 4.84 (3.08, 7.77), 4.84 (1.40, 18.34), 3.75 (1.98, 7.12), 4.22 (1.21, 15.97), and 2.33 (1.25, 4.37), respectively (Table 2). The combination of Compound kushen injection and radiotherapy led to the best performance status, with a SUCRA value of 78.05% (Figure 4B). The SUCRA values of the other CHIs were listed in Table 3.

**Figure 5:**
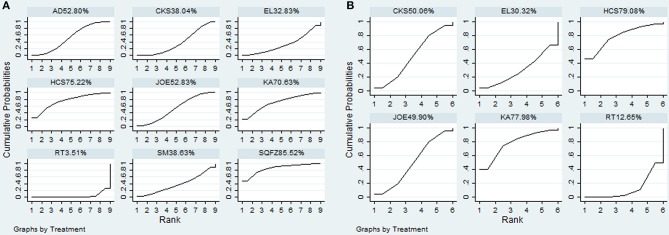
SUCRA for the treatments of 1-year survival rate **(A)** and 2-year survival rate **(B)**.

Additionally, 20 RCTs with 8 types of CHIs contributed to the evidence network for 1-year survival rate analysis. Aidi, Shenqifuzheng, Compound kushen, Huachansu, Kangai, and Javanica oil emulsion injection combined with radiotherapy were associated with a 1-year-longer survival rate than radiotherapy alone. Their ORs and 95% CIs were 0.40 (0.26, 0.60), 5.6 (1.67, 20.68), 2.04 (1.26, 3.30), 4.11 (1.32, 14.66), 3.77 (1.14, 14.34), and 2.52 (1.47, 4.32), respectively (Table 4). The SUCRA values suggested that Shenqifusheng injection (85.52%) was the best choice for this outcome. With regard to the 2-year survival rate, 9 RCTs with 5 types of CHIs described the data, and the combination of Huachansu, Kangai, Compound kushen, Javanica oil emulsion, and Elemene injection with radiotherapy had no corresponding benefit for improving the 2-year survival rate (Figure 5).

**Table 4:** Results of the network meta-analysis for 1-year survival rate (upper-right quadrant) and 2-year survival rate (lower-left quadrant).

**AD+RT**	**0.40** **(0.26, 0.60)**	0.77 (0.23, 2.74)	2.22 (0.61, 8.77)	0.81 (0.43, 1.54)	1.64 (0.49, 6.19)	1.50 (0.42, 6.05)	0.71 (0.24, 2.12)	1.00 (0.51, 1.98)
–	**RT**	1.95 (0.61, 6.53)	**5.6** **(1.67, 20.68)**	**2.04** **(1.26, 3.30)**	**4.11** **(1.32, 14.66)**	**3.77** **(1.14, 14.34)**	1.78 (0.65, 4.93)	**2.52** **(1.47, 4.32)**
–	–	**SM+RT**	2.87 (0.51, 16.38)	1.05 (0.29, 3.63)	2.11 (0.41, 11.4)	1.94 (0.33, 11.41)	0.90 (0.19, 4.22)	1.30 (0.35, 4.65)
–	–	–	**SQFZ+RT**	0.37 (0.092, 1.34)	0.73 (0.14, 4.07)	0.68 (0.11, 4.02)	0.31 (0.061, 1.59)	0.45 (0.11, 1.74)
–	0.56 (0.21, 1.55)	–	–	**CKS+RT**	2.04 (0.57, 7.81)	1.85 (0.51, 7.66)	0.87 (0.29, 2.72)	1.24 (0.61, 2.58)
–	3.76 (0.65, 23.12)	–	–	2.10 (0.28, 16.7)	**HCS+RT**	0.90 (0.16, 5.41)	0.43 (0.087, 1.93)	0.61 (0.16, 2.11)
–	3.50 (0.64, 18.97)	–	–	1.95 (0.28, 14.22)	0.93 (0.078, 10.62)	**KA+RT**	0.47 (0.090, 2.29)	0.67 (0.16, 2.51)
–	1.24 (0.23, 6.52)	–	–	0.69 (0.10, 4.91)	0.33 (0.029, 3.72)	0.35 (0.033, 3.84)	**EL+RT**	1.43 (0.44, 4.44)
–	1.77 (0.72, 5.32)	–	–	0.99 (0.27, 4.61)	0.47 (0.065, 3.90)	0.51 (0.078, 4.00)	1.43 (0.23, 10.99)	**JOE+RT**

### Safety Outcome

**Figure 6:**
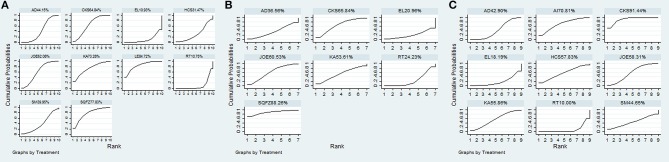
SUCRA for the treatments of ADRs. **(A)** leucopenia; **(B)** gastrointestinal reactions; **(C)** radiation esophagitis.

A total of 18 RCTs with 9 CHIs were involved in the NMA concerning leucopenia. The results indicated a favorable trend for relieving leucopenia when Aidi, Shenqifuzheng, Compound kushen, Kangai, Lentinan, and Javanica oil emulsion injection were used during radiotherapy; their ORs and 95% CIs were 2.78 (1.42, 5.33), 0.13 (0.026, 0.63), 0.22 (0.078, 0.56), 0.15 (0.015, 0.96), 0.098 (0.014, 0.53), and 0.23 (0.10, 0.51), respectively. Compared with Elemene injection, Shenqifuzheng and Lentinan injection might hold greater potential for relieving leucopenia, with ORs and 95% CIs of 8.80 (1.04, 78.73) and 0.081 (0.0078, 0.77), respectively (Table 5). The results of the SUCRA analysis showed that Lentinan injection was superior to all other candidate interventions for decreasing the risk of leucopenia (Figure 6A).

**Table 5:** Results of the network meta-analysis for leucopenia (upper-right quadrant) and gastrointestinal reactions (lower-left quadrant).

**AD+RT**	**2.78** **(1.42, 5.33)**	1.18 (0.23, 5.80)	0.37 (0.063, 1.97)	0.61 (0.18, 1.91)	1.56 (0.27, 8.86)	0.41 (0.036, 3.02)	3.27 (0.66, 15.97)	0.27 (0.035, 1.65)	0.65 (0.23, 1.81)
1.20 (0.14, 10.34)	**RT**	0.42 (0.097, 1.82)	**0.13** **(0.026, 0.63)**	**0.22** **(0.078, 0.56)**	0.56 (0.11, 2.80)	**0.15** **(0.015, 0.96)**	1.17 (0.27, 5.02)	**0.098** **(0.014, 0.53)**	**0.23** **(0.10, 0.51)**
–	–	**SM+RT**	0.31 (0.035, 2.68)	0.51 (0.086, 2.95)	1.32 (0.14, 11.65)	0.34 (0.023, 3.82)	2.76 (0.35, 22.06)	0.23 (0.021, 2.21)	0.55 (0.10, 2.90)
0.16 (0.0051, 4.32)	0.14 (0.0085, 1.64)	–	**SQFZ+RT**	1.63 (0.25, 10.62)	4.17 (0.44, 42.52)	1.09 (0.069, 13.29)	**8.80** **(1.04, 78.73)**	0.72 (0.063, 7.76)	1.75 (0.30, 10.9)
0.50 (0.041, 6.68)	0.42 (0.11, 1.69)	–	3.12 (0.18, 68.41)	**CKS+RT**	2.57 (0.40, 17.51)	0.66 (0.056, 5.84)	5.39 (0.96, 32.27)	0.45 (0.053, 3.26)	1.08 (0.31, 3.93)
–	–	–	–	–	**HCS+RT**	0.26 (0.016, 3.18)	2.10 (0.24, 18.33)	0.17 (0.014, 1.84)	0.42 (0.068, 2.54)
0.66 (0.031, 14.22)	0.55 (0.061, 4.87)	–	4.05 (0.14, 135.2)	1.31 (0.096, 17.09)	–	**KA+RT**	8.12 (0.73, 119.5)	0.67 (0.045, 11.36)	1.61 (0.21, 18.15)
1.54 (0.075, 33.54)	1.28 (0.15, 11.36)	–	9.48 (0.35, 311.4)	3.06 (0.23, 40.49)	–	2.35 (0.11, 52)	**EL+RT**	**0.081** **(0.0078, 0.77)**	0.20 (0.038, 1.04)
–	–	–	–	–	–	–	–	**LE+RT**	2.42 (0.37, 18.97)
0.58 (0.043, 6.53)	0.48 (0.12, 1.69)	–	3.51 (0.19, 72.43)	1.15 (0.15, 7.05)	–	0.88 (0.062, 10.55)	0.37 (0.027, 4.33)	–	**JOE+RT**

Ten RCTs with 6 CHIs were included in the NMA concerning gastrointestinal reactions. Table 5 presents evidence that the combination of different CHIs did not reduce the risk of gastrointestinal reactions compared with radiotherapy alone. The superiority of Shenqifuzheng injection over other types of CHIs in relieving gastrointestinal reactions was further confirmed via SUCRA analysis (Figure 6B).

Data on radiation esophagitis were available for 33 trials involving 8 CHIs. The results suggested that the combination of Aidi, Compound kushen, Astragalus polysaccharide, and Javanica oil emulsion injection with radiotherapy displayed a trend of being beneficial for relieving radiation esophagitis; their ORs and 95% CIs were 1.76 (1.23, 2.56), 0.24 (0.15, 0.38), 0.35 (0.13, 0.96), and 0.45 (0.27, 0.76). Among the CHIs, Compound kushen injection yielded the best result for reducing radiation esophagitis compared with Aidi and Elemene injection; the ORs and 95% CIs were 0.42 (0.23, 0.75) and 3.86 (1.48, 10.2) (Table 6). The SUCRA results showed that Compound kushen injection was regarded as more efficient in relieving radiation esophagitis than other types of CHIs (Figure 6C).

**Table 6:** Results of the network meta-analysis for radiation esophagitis.

**AD+RT**								
**1.76** **(1.23, 2.56)**	**RT**							
1.02 (0.191, 4.91)	0.57 (0.11, 2.69)	**SM+RT**						
**0.42** **(0.23, 0.75)**	**0.24** **(0.15, 0.38)**	0.41 (0.083, 2.26)	**CKS+RT**					
0.78 (0.22, 2.60)	0.44 (0.13, 1.33)	0.77 (0.11, 5.41)	1.87 (0.51, 6.12)	**HCS+RT**				
0.62 (0.21, 1.77)	**0.35** **(0.13, 0.96)**	0.61 (0.096, 3.97)	1.46 (0.49, 4.52)	0.79 (0.19, 3.82)	**AI+RT**			
0.81 (0.32, 2.08)	0.46 (0.19, 1.09)	0.80 (0.14, 5.12)	1.94 (0.73, 5.14)	1.06 (0.24, 4.62)	1.32 (0.36, 4.91)	**KA+RT**		
1.63 (0.64, 4.09)	0.92 (0.39, 2.15)	1.61 (0.28, 10.11)	**3.86** **(1.48, 10.2)**	2.09 (0.51, 9.15)	2.64 (0.71, 9.92)	1.98 (0.60, 6.80)	**EL+RT**	
0.80 (0.42, 1.49)	**0.45** **(0.27, 0.76)**	0.79 (0.15, 4.27)	1.89 (0.94, 3.8)	1.02 (0.30, 3.81)	1.30 (0.41, 3.99)	0.98 (0.35, 2.70)	0.49 (0.18, 1.34)	**JOE+RT**

### Publication Bias

**Figure 7:**
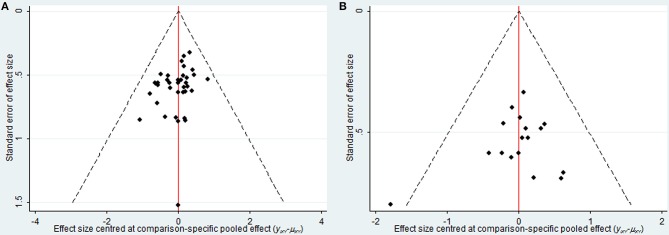
Funnel plot of clinical effectiveness rate **(A)** and performance status **(B)**.

As depicted in Figure 7, the publication bias of the included trials regarding the clinical effectiveness rate and performance status were measured by funnel plots. The results illustrated potential publication bias among included RCTs.

### Cluster Analysis

**Figure 8:**
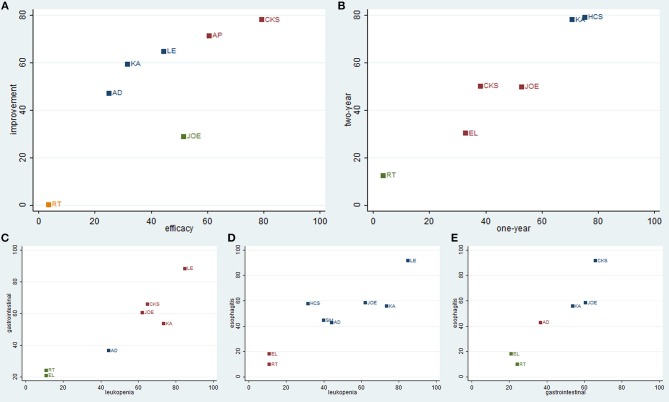
Cluster analysis plot of outcomes. **(A)** clinical effectiveness rate (X axis) and performance status (Y axis); **(B)** 1-year survival rate (X axis) and 2-year survival rate (Y axis); **(C)** leucopenia (X axis) and gastrointestinal reactions (Y axis); **(D)** leucopenia (X axis) and radiation esophagitis (Y axis); **(E)** gastrointestinal reactions (X axis) and radiation esophagitis (Y axis).

To categorize the different types of CHIs into distinctive groups and estimate the safest and most effective treatments, we conducted a cluster analysis for RCTs that described the details of several outcomes simultaneously. Regarding the efficacy outcomes, the results of the cluster analysis revealed (as shown in Figure 8) that Compound kushen injection was associated with the most favorable benefits in improving the clinical effectiveness rate and performance status compared with other CHIs. Huachansu and Kangai injection were superior in improving 1-year and 2-year survival rates. With respect to safety outcomes, Lentinan injection had a clear therapeutic advantage for reliving ADRs, and Compound kushen injection achieved superior effects for reducing both gastrointestinal reaction and radiation esophagitis among the CHIs. Overall, the combination of Compound kushen injection and radiotherapy had the potential to be the most preferable and beneficial option for patients with esophageal cancer in terms of efficacy and safety.

## Discussion

To compare the efficacy and safety of different CHIs combined with radiotherapy to treat esophageal cancer, we adopt the NMA approach to analyze the overwhelming evidence in published RCTs. In summary, the results of the NMA indicated that the combination of Compound kushen injection and radiotherapy was the best choice for patients with esophageal cancer in terms of both efficacy and safety. Moreover, the choice of specific CHIs should rely on high-quality evidence-based research, the clinical practice of oncologists and the condition of patients with esophageal cancer.

It has been proven that esophageal cancer is still difficult to cure despite improved surgical techniques, reduced perioperative mortality, and the introduction of multimodality therapy (Dehdashti and Siegel, 2004; Kato et al., 2007; Kato and Nakajima, 2012). TCM is a widely popular treatment for esophageal cancer because of its benefits for syndrome differentiation, specimen and centralizer treatment. It had advantages in suppressing tumor progression, killing tumor cells, improving the sensitivity of chemotherapy or radiotherapy, and improving immunologic functions (Wang et al., 2014; Zhang et al., 2014). As a TCM preparation, Compound kushen injection is derived from *Radix sophorae flavescentis* and *Rhizoma smilacis glabrae*, and it has been approved for treating various types of solid tumors by the State Food and Drug Administration of China for over 20 years (Guo et al., 2015; Gao et al., 2018). It is well documented that Compound kushen injection can offer clear advantages for patients with liver cancer, colon cancer, lung cancer, and acute leukemia, according to several systematic reviews (Ma et al., 2016; Tu et al., 2016; Wang et al., 2016; Yu et al., 2017; Zhang et al., 2017a). The molecular mechanism of Compound kushen injection against esophageal cancer remarkably enhances the expression of pro-apoptotic gene TE-8 cells through increasing apoptosis and inhibiting cell proliferation (Yang et al., 2015). Huanchansu injection is the sterilized hot water extraction of dried toad skin. Previous studies have confirmed that its anticancer potentials inhibit the proliferation and differentiation of tumor cells and enhance immune responses (Qi et al., 2014; Wei et al., 2017). The main constituents of Kangai injection include Astragalus saponins, ginsenoside, and matrine that are isolated from Chinese herbs, namely, *ginseng, Astragalus*, and *Sophora flavescens*. These active constituents play an important regulatory role in the tumor development process (Auyeung et al., 2009; Yong et al., 2015). Lentinan injection, the backbone of β-(1, 3)-glucan with β-(1, 6) branches, has also been administered for antitumor therapy in China and Japan for decades. Correlative studies have revealed the properties of Lentinan injection in relation to immunomodulation and improvements in the response rate as well as adverse events in advanced cancer treatment (Ina et al., 2013; Wang et al., 2017). With regard to CHI safety, our results revealed that some types of CHIs elicited a greater beneficial impact on reliving ADRs (leucopenia, gastrointestinal reactions, and radiation esophagitis) than radiotherapy alone. However, the safety of CHIs should be adequately addressed and considered for safe practice in accordance with the specific environment, especially in developing countries, where the unsafe and excessive use of CHIs is common in health care systems.

Currently, only one published NMA has focused on CHIs combined with radiotherapy for treating esophageal cancer (Ge et al., 2015). The search period of the previous systematic review ended in September 2014, and it included 43 RCTs involving 3,289 participants. By contrast, the strengths of the present study include the comprehensive coverage of the current and latest research findings, and the inclusion criteria were formulated and established strictly through the selection process of potential RCTs. For example, the control group of the included RCTs was restricted to studies in which patients only received radiotherapy in order to reduce the interference of clinical heterogeneity. Meanwhile, the strong and robust Bayesian NMA statistical method was utilized to evaluate the different efficacy and safety outcomes. The efficacy outcomes in the present study involved the clinical effectiveness rate, performance status, and 1-year and 2-year survival rates; the safety outcomes were ADRs, such as leucopenia, gastrointestinal reactions, and radiation esophagitis. SUCRA was used to identify the optimal treatment for each outcome, and a cluster analysis was performed to estimate the superior CHIs in terms of efficacy and safety.

Several limitations of the current NMA should be noted. First, as mentioned above, there was a lack of head-to-head comparisons of different CHIs, although this limitation was addressed and overcome via NMA to a certain extent. More head-to-head comparisons for different types of CHIs are warranted to draw more robust and reliable conclusions. Second, the endpoint outcomes, such as overall survival and progression-free survival, play a vital role in identifying and judging the therapeutic effects among patients with tumors, while the majority of the included trials did not report long-term endpoint outcomes. Hence, clinical trials of patients with cancer should focus on more meaningful endpoints. Last, some confounding factors were inherent in the included trials; for instance, the cancer stages as well as the doses and courses of CHIs varied across the included RCTs. In addition, the majority of the included RCTs exhibited a high risk of bias, and the sample size in each study was relatively small. Another important limitation of the present meta-analysis relates to the methodological quality of the included RCTs. It would be desirable for trials to provide details in regard to the randomized method, allocation concealment, and blind methods used because the methodological quality of the included RCTs was closely associated with the credibility of the evaluation results in the systematic review. For this reason, we suggest that clinical trials should pay attention to improving the methodological quality in order to support and promote the appropriate use of CHIs. Given the limited quality and quantity of the included studies, more rigorous RCTs are needed to verify the beneficial role of CHIs combined with radiotherapy in patients with esophageal cancer.

In conclusion, the current evidence suggests that the combination of Compound kushen injection and radiotherapy is the most preferable and beneficial option for patients with esophageal cancer in terms of efficacy and safety, although additional results from multi-center trials and high-quality studies will be pivotal for supporting our findings.

## Author Contributions

DZ, MN, JW, SL, and ZM: conception and design. DZ, MN, JW, SL, JT, and BZ: development of methodology. DZ, MN, JW, and SL: acquisition of data. DZ, MN, JW, SL, JT, and BZ: analysis and interpretation of data. DZ, MN, and JW: writing, review, or revision of the manuscript. DZ, MN, and JT: administrative, technical, and material support. DZ, MN, JW, ZM, SL, XZ, JT, and BZ: study supervision.

### Conflict of Interest Statement

The authors declare that the research was conducted in the absence of any commercial or financial relationships that could be construed as a potential conflict of interest.

## Supplementary Material

Table S1 Search strategy.

Table S2 PRISMA flow diagram.

Table S3 PRISMA checklist.

Figure S1 Risk-of-bias summary.

File S1 Included RCTs.

File S2 Quality evaluation of the included RCTs.

## Abbreviations

AD: Aidi injection
AI: Astragalus injection
AP: Astragalus polysaccharide injection
CBMdisc: China Biology Medicine Disc
CHIs: Chinese herbal injections
CI: confidence interval
CKS: Compound kushen injection
CNKI: China National Knowledge Infrastructure Database
DC: Disodium cantharidinate and vitamin B6 injection
EL: Elemene injection
HCS: Huachansu injection
JOE: Javanica oil emulsion injection
KA: Kangai injection
KPS: Karnofsky performance score
LE: Lentinan injection
L-OHP: oxaliplatin
NMA: network meta-analysis
OR: odds ratios
RCTs: randomized controlled trials
SM: Shenmai injection
SQFZ: Shenqifuzheng injection
SUCRA: Surface under the cumulative ranking probabilities
TCM: Traditional Chinese medicine
VIP: Cqvip Database
WHO: World Health Organization.

## References

[B1] Achana F. A. Cooper N. J. Bujkiewicz S. Hubbard S. J. Kendrick D. Jones D. R.. (2014). Network meta-analysis of multiple outcome measures accounting for borrowing of information across outcomes. BMC Med. Res. Methodol. 14:92. 10.1186/1471-2288-14-92 25047164 PMC4142066

[B2] Auyeung K. K. Cho C. H. Ko J. K. (2009). A novel anticancer effect of astragalus saponins: transcriptional activation of NSAID-activated gene. Int. J. Cancer 125, 1082–1091. 10.1002/ijc.24397 19384947

[B3] Beukema J. C. Van L. P. Widder J. Langendijk J. A. Muijs C. T. (2015). Is cardiac toxicity a relevant issue in the radiation treatment of esophageal cancer?. Radiother. Oncol. J. Eur. Soc. Ther. Radiol. Oncol. 114, 85–90. 10.1016/j.radonc.2014.11.037 25554226

[B4] Cai Y. M. Zhu H. Niu J. X. Bing L. Sun Z. Zhang W. H.. (2017). Identification of herb pairs in esophageal cancer. Complement. Med. Res. 24, 40–45. 10.1159/000454699 28219055

[B5] Cappetta A. Lonardi S. Pastorelli D. Bergamo F. Lombardi G. Zagonel V. (2012). Advanced gastric cancer (GC) and cancer of the gastro-oesophageal junction (GEJ): focus on targeted therapies. Crit. Rev. Oncol. Hematol. 81, 38–48. 10.1016/j.critrevonc.2010.12.006 21256046

[B6] Chaimani A. Higgins J. P. Mavridis D. Spyridonos P. Salanti G. (2013). Graphical tools for network meta-analysis in STATA. PLoS ONE 10:e76654 10.1371/journal.pone.0076654 PMC3789683 24098547

[B7] Chan J. S. (2016). Bayesian informative dropout model for longitudinal binary data with random effects using conditional and joint modeling approaches. Biom. J. 58, 549–569. 10.1002/bimj.201400064 26467236

[B8] Chung V. C. Wu X. Hui E. P. Ziea E. T. Ng B. F. Ho R. S.. (2015). Effectiveness of Chinese herbal medicine for cancer palliative care: overview of systematic reviews with meta-analyses. Sci. Rep. 5:18111. 10.1038/srep18111 26669761 PMC4680970

[B9] Dehdashti F. Siegel B. A. (2004). Neoplasms of the esophagus and stomach. Semin. Nucl. Med. 34, 198–208. 10.1053/j.semnuclmed.2004.03.005 15202101

[B10] Donegan S. Williamson P. D'Alessandro U. Tudur S. C. (2013). Assessing key assumptions of network meta-analysis: a review of methods. Res. Synth. Methods 4, 291–323. 10.1002/jrsm.1085 26053945

[B11] Ferlay J. Soerjomataram I. Dikshit R. Eser S. Mathers C. Rebelo M.. (2015). Cancer incidence and mortality worldwide: sources, methods and major patterns in GLOBOCAN 2012. Int. J. Cancer 136, E359–386. 10.1002/ijc.29210 25220842

[B12] Gao L. Wang K. X. Zhou Y. Z. Fang J. S. Qin X. M. Du G. H. (2018). Uncovering the anticancer mechanism of Compound Kushen Injection against HCC by integrating quantitative analysis, network analysis and experimental validation. Sci. Rep. 8:624. 10.1038/s41598-017-18325-7 29330507 PMC5766629

[B13] Ge L. Mao L. Tian J. H. Shi F. Y. Lou L. L. Qiu X.. (2015). Network meta-analysis on selecting Chinese medical injections in radiotherapy for esophageal cancer. China J. Chin. Mater. Med. 40, 3674–3681 26983220

[B14] Greco T. Landoni G. Biondi-Zoccai G. D'Ascenzo F. Zangrillo A. (2016). A Bayesian network meta-analysis for binary outcome: how to do it. Stat. Methods Med. Res. 5, 1757–1773. 10.1177/0962280213500185 23970014

[B15] Guo Y. M. Huang Y. X. Shen H. H. Sang X. X. Ma X. Zhao Y. L.. (2015). Efficacy of compound kushen injection in relieving cancer-related pain: a systematic review and meta-analysis. Evid. Based Complement. Alternat. Med. 2015:840742. 10.1155/2015/840742 26504481 PMC4609400

[B16] Higgins J. P. Altman D. G. Gøtzsche P. C. Jüni P. Moher D. Oxman A. D.. (2011). The Cochrane Collaboration's tool for assessing risk of bias in randomised trials. BMJ 343:d5928. 10.1136/bmj.d5928 22008217 PMC3196245

[B17] Ina K. Kataoka T. Ando T. (2013). The use of lentinan for treating gastric cancer. Anticancer Agents Med. Chem. 13, 681–688. 10.2174/1871520611313050002 23092289 PMC3664515

[B18] Jackson D. Turner R. Rhodes K. Viechtbauer W. (2014). Methods for calculating confidence and credible intervals for the residual between-study variance in random effects meta-regression models. BMC Med. Res. Methodol. 14:103. 10.1186/1471-2288-14-103 25196829 PMC4160560

[B19] Kato H. Fukuchi M. Miyazaki T. Nakajima M. Tanaka N. Inose T.. (2007). Surgical treatment for esophageal cancer. Dig. Surg. 24, 88–95. 10.1159/000101894 17446703

[B20] Kato H. Nakajima M. (2012). The efficacy of fdg-pet for the management of esophageal cancer: review article. Ann. Thorac. Cardiovasc. Surg. Off J. Assoc. Thorac. Cardiovasc. Surge Asia 18:412. 10.5761/atcs.ra.12.01954 22785452

[B21] Krahn U. Binder H. König J. (2014). Visualizing inconsistency in network meta-analysis by independent path decomposition. BMC Med. Res. Methodol 14:131. 10.1186/1471-2288-14-131 25510877 PMC4279676

[B22] Laws A. Kendall R. Hawkins N. (2014). A comparison of national guidelines for network meta-analysis. Value Health 17, 642–654. 10.1016/j.jval.2014.06.001 25128059

[B23] Liberati A. Altman D. G. Tetzlaff J. Mulrow C. Gøtzsche P. C. Ioannidis J. P.. (2009). The PRISMA statement for reporting systematic reviews and meta-analyses of studies that evaluate health care interventions: explanation and elaboration. PLoS Med. 6:e1000100. 10.1371/journal.pmed.1000100 19621070 PMC2707010

[B24] Lin Y. Totsuka Y. He Y. Kikuchi S. Qiao Y. Ueda J.. (2013). Epidemiology of esophageal cancer in Japan and China. J. Epidemiol. 23, 233–242. 10.2188/jea.JE20120162 23629646 PMC3709543

[B25] Ma X. Li R. S. Wang J. Huang Y. Q. Li P. Y. Wang J.. (2016). The therapeutic efficacy and safety of compound kushen injection combined with transarterial chemoembolization in unresectable hepatocellular carcinoma: an update systematic review and meta-analysis. Front. Pharmacol. 7:70. 10.3389/fphar.2016.00070 27065861 PMC4814457

[B26] Mavridis D. Welton N. J. Sutton A. Salanti G. (2014). A selection model for accounting for publication bias in a full network meta-analysis. Stat. Med. 30, 5399–53412. 10.1002/sim.6321 25316006

[B27] Nguyen N. P. Jang S. Vock J. Vinh-Hung V. Chi A. Vos P.. (2014). Feasibility of intensity-modulated and image-guided radiotherapy for locally advanced esophageal cancer. BMC Cancer 14:265. 10.1186/1471-2407-14-265 24742268 PMC3996254

[B28] Pennathur A. Gibson M. K. Jobe B. A. Luketich J. D. (2013). Oesophageal carcinoma. Lancet 381, 400–412. 10.1016/S0140-6736(12)60643-6 23374478

[B29] Piepho H. P. (2014). Network-meta analysis made easy: detection of inconsistency using factorial analysis-of-variance models. BMC Med. Res. Methodol. 14:61. 10.1186/1471-2288-14-61 24885590 PMC4049370

[B30] Qi J. Tan C. K. Hashimi S. M. Zulfiker A. H. Good D. Wei M. Q. (2014). Toad glandular secretions and skin extractions as anti-inflammatory and anticancer agents. Evid. Based Complement. Alternat. Med. 2014:312684. 10.1155/2014/312684 24734105 PMC3963377

[B31] Rücker G. Schwarzer G. (2015). Ranking treatments in frequentist network meta-analysis works without resampling methods. BMC Med. Res. Methodol. 15:58. 10.1186/s12874-015-0060-8 26227148 PMC4521472

[B32] Salanti G. Del G. C. Chaimani A. Caldwell D. M. Higgins J. P. (2014). Evaluating the quality of evidence from a network meta-analysis. PLoS ONE 9:e99682. 10.1371/journal.pone.0099682 24992266 PMC4084629

[B33] Shim S. Yoon B. H. Shin I. S. Bae J. M. (2017). Network meta-analysis: application and practice using Stata. Epidemiol. Health 39:e2017047. 10.4178/epih.e2017047 29092392 PMC5733388

[B34] Shridhar R. Almhanna K. Meredith K. L. Biagioli M. C. Chuong M. D. Cruz A.. (2013). Radiation therapy and esophageal cancer. Cancer Control 20, 97–110. 10.1177/107327481302000203 23571700

[B35] Stephenson M. Fleetwood K. Yellowlees A. (2015). Alternatives to Winbugs for network meta-analysis. Value Health 7:A720 10.1016/j.jval.2015.09.2730

[B36] Trinquart L. Attiche N. Bafeta A. Porcher R. Ravaud P. (2016). Uncertainty in treatment rankings: reanalysis of network meta-analyses of randomized trials. Ann. Intern. Med. 10, 666–673. 10.7326/M15-2521 27089537

[B37] Trinquart L. Chatellier G. Ravaud P. (2012). Adjustment for reporting bias in network meta-analysis of antidepressant trials. BMC Med. Res. Methodol. 12:150. 10.1186/1471-2288-12-150 23016799 PMC3537713

[B38] Tu H. Lei B. Meng S. Liu H. Wei Y. He A. (2016). Efficacy of compound kushen injection in combination with induction chemotherapy for treating adult patients newly diagnosed with acute leukemia. Evid. Based Complement. Alternat. Med. 1, 1–7. 10.1155/2016/3121402 PMC5050378 27738441

[B39] Veroniki A. A. Soobiah C. Tricco A. C. Elliott M. J. Straus S. E. (2015). Methods and characteristics of published network meta-analyses using individual patient data: protocol for a scoping review. BMJ Open 5:e007103. 10.1136/bmjopen-2014-007103 25926144 PMC4420933

[B40] Wang C. Y. Bai X. Y. Wang C. H. (2014). Traditional Chinese medicine: a treasured natural resource of anticancer drug research and development. Am. J. Chin. Med. 42, 543–559. 10.1142/S0192415X14500359 24871650

[B41] Wang H. Cai Y. Zheng Y. Bai Q. Xie D. Yu J. (2017). Efficacy of biological response modifier lentinan with chemotherapy for advanced cancer: a meta-analysis. Cancer Med. 6, 2222–2233. 10.1002/cam4.1156 28940986 PMC5633561

[B42] Wang J. B. Fan J. H. Liang H. Li J. Xiao H. J. Wei W. Q.. (2012). Attributable causes of esophageal cancer incidence and mortality in China. PLoS ONE 7:e42281. 10.1371/journal.pone.0042281 22876312 PMC3410925

[B43] Wang Q. Chen D. Y. (2009). Effect of Aidi injection on peripheral blood expression of Th1/Th2 transcription factors and cytokines in patients with esophageal squamous cell carcinoma during radiotherapy. Chin. J. Integ. Tradit. Western Med. 29, 394–397. 19673326

[B44] Wang S. Lian X. Sun M. Luo L. Guo L. (2016). Efficacy of compound kushen injection plus radiotherapy on nonsmall-cell lung cancer: a systematic review and meta-analysis. J. Cancer Res. Ther. 12, 1298–1306. 10.4103/0973-1482 28169243

[B45] Wei X. Si N. Zhang Y. Zhao H. Yang J. Wang H.. (2017). Evaluation of bufadienolides as the main antitumor components in cinobufacin injection for liver and gastric cancer therapy. PLoS ONE 12:e0169141. 10.1371/journal.pone.0169141 28081155 PMC5231367

[B46] World Health Organization (1979). W. H. O. Handbook for Reporting Results of Cancer Treatment. Available online at: http://apps.who.int/iris/handle/10665/37200. (Accessed June 1, 2017).

[B47] Wu X. Chung V. C. Lu P. Poon S. K. Hui E. P. Lau A. Y.. (2016). Chinese herbal medicine for improving quality of life among nonsmall cell lung cancer patients: overview of systematic reviews and network meta-analysis. Medicine 95:e2410. 10.1097/MD.0000000000002410 26735544 PMC4706264

[B48] Xu Z. Xu C. Ge H. Li Y. Chu L. Zhang J.. (2015). Modified dachengqi tang improves decreased gastrointestinal motility in postoperative esophageal cancer patients. J. Tradit. Chin. Med. 35, 249–254 26237826 10.1016/s0254-6272(15)30093-5

[B49] Yang X. Cai W. Yang Q. Zhihong L. U. Jinsong L. I. Jian Y. U. (2015). Compound radix sophorae flavescentis exerts antitumor effects by inhibiting the proliferation and inducing the apoptosis of esophageal carcinoma te-8 cells. Oncol. Lett. 10, 2209–2213. 10.3892/ol.2015.3607 26622820 PMC4579809

[B50] Yong J. Wu X. Lu C. (2015). Anticancer advances of matrine and its derivatives. Curr. Pharm. Des. 21, 3673–3680. 25613788 10.2174/1381612821666150122123748

[B51] Yu L. Zhou Y. Yang Y. Lu F. Fan Y. (2017). Efficacy and safety of compound kushen injection on patients with advanced colon cancer: a meta-analysis of randomized controlled trials. Evid. Based Complement. Alternat. Med. 7269, 1–9. 10.1155/2017/7102514 PMC5702402 29259647

[B52] Zacherl J. (2014). The current evidence in support of multimodal treatment of locally advanced, potentially resectable esophageal cancer. Dig. Dis. 32, 171–175. 10.1159/000357189 24603404

[B53] Zhang D. Zheng J. Ni M. Wu J. Wang K. Duan X.. (2017b). Comparative efficacy and safety of Chinese herbal injections combined with the FOLFOX regimen for treating gastric cancer in China: a network meta-analysis. Oncotarget 8, 68873–68889. 10.18632/oncotarget.20320 28978164 PMC5620304

[B54] Zhang L. Ma J. Han Y. Liu J. Zhou W. Hong L.. (2016). Targeted therapy in esophageal cancer. Expert. Rev. Gastroenterol. Hepatol. 10, 595–604. 10.1586/17474124.2016.1140036 26895097

[B55] Zhang L. Wu C. Zhang Y. Liu F. Wang X. Zhao M.. (2014). Comparison of efficacy and toxicity of traditional Chinese medicine (TCM) herbal mixture LQ and conventional chemotherapy on lung cancer metastasis and survival in mouse models. PLoS ONE 9:e109814.e109836. 10.1371/journal.pone.0109814 25286158 PMC4186882

[B56] Zhang Y. Hui F. Yang Y. Chu H. Qin X. Zhao M.. (2017a). Can kushen injection combined with TACE improve therapeutic efficacy and safety in patients with advanced HCC? A systematic review and network meta-analysis. Oncotarget 8, 107258–107272. 10.18632/oncotarget.20921 29291026 PMC5739811

