# Supplementary material for: The Optimal Chinese Herbal Injections for Use With Radiotherapy to Treat Esophageal Cancer: A Systematic Review and Bayesian Network Meta-Analysis
Source: Front Pharmacol. 2019 Jan 4;9:1470. doi: 10.3389/fphar.2018.01470 (PMC6329258; doi:10.3389/fphar.2018.01470)
Supplement: Table S1 — Search strategy. [file Table_1.pdf]

Supplementary Material

Are there the optimal Chinese herbal injections plus radiotherapy for esophageal cancer? A systematic review and Bayesian network meta-analysis.

Dan Zhang[1], Meng-Wei Ni[1], Jia-Rui Wu[1], Shu-Yu Liu[1], Zi-Qi Meng[1], Jin-Hui Tian[2], Xiao-Meng Zhang[1] and Bing Zhang[1]

PRISMA checklist for network meta-analysis:

Section/topic	#	Checklist item	Reported on page #
**TITLE**			
Title	1	Identify the report as a systematic review incorporating a network meta-analysis (or related form of meta-analysis).	1
**ABSTRACT**			
Structured summary	2	Provide a structured summary including, as applicable:<br>Background: main objectives<br>Methods: data sources; study eligibility criteria, participants, and interventions; study appraisal; and synthesis methods, such as network meta-analysis.<br>Results: number of studies and participants identified; summary estimates with corresponding confidence/credible intervals; treatment rankings may also be discussed. Authors may choose to summarize pairwise comparisons against a chosen treatment included in their analyses for brevity.<br>Discussion/Conclusions: limitations; conclusions and implications of findings.<br>Other: primary source of funding; systematic review registration number with registry name.	2
**INTRODUCTION**			
Rationale	3	Describe the rationale for the review in the context of what is already known, including mention of why a network meta-analysis has been conducted	2-3
Objectives	4	Provide an explicit statement of questions being addressed with reference to participants, interventions, comparisons, outcomes, and study design (PICOS).	2-3
**METHODS**			
Protocol and registration	5	Indicate if a review protocol exists and if and where it can be accessed (e.g., Web address), and, if available, provide registration information including registration number.	/

Eligibility criteria	6	Specify study characteristics (e.g., PICOS, length of follow-up) and report characteristics (e.g., years considered, language, publication status) used as criteria for eligibility, giving rationale. Clearly describe eligible treatments included in the treatment network, and note whether any have been clustered or merged into the same node (with justification).	3-4
Information sources	7	Describe all information sources (e.g., databases with dates of coverage, contact with study authors to identify additional studies) in the search and date last searched.	3-4
Search	8	Present full electronic search strategy for at least one database, including any limits used, such that it could be repeated.	3-4
Study selection	9	State the process for selecting studies (i.e., screening, eligibility, included in systematic review, and, if applicable, included in the meta-analysis).	4
Data collection process	10	Describe method of data extraction from reports (e.g., piloted forms, independently, in duplicate) and any processes for obtaining and confirming data from investigators.	4-5
Data items	11	List and define all variables for which data were sought (e.g., PICOS, funding sources) and any assumptions and simplifications made.	4-5
Geometry of the network	12	Describe methods used to explore the geometry of the treatment network under study and potential biases related to it. This should include how the evidence base has been graphically summarized for presentation, and what characteristics were compiled and used to describe the evidence base to readers	5
Risk of bias within individual studies	13	Describe methods used for assessing risk of bias of individual studies (including specification of whether this was done at the study or outcome level), and how this information is to be used in any data synthesis.	5
Summary measures	14	State the principal summary measures (e.g., risk ratio, difference in means). Also describe the use of additional summary measures assessed, such as treatment rankings and surface under the cumulative ranking curve (SUCRA) values, as well as modified approaches used to present summary findings from meta-analyses.	5-6
Planned methods of analysis	15	Describe the methods of handling data and combining results of studies for each network meta-analysis. This should include, but not be limited to: Handling of multigroup trials; Selection of variance structure; Selection of prior distributions in Bayesian analyses; and Assessment of model fit.	5-6
Assessment of inconsistency	16	Describe the statistical methods used to evaluate the agreement of direct and indirect evidence in the treatment network(s) studied. Describe efforts taken to address its presence when found.	6
Risk of bias across studies	17	Specify any assessment of risk of bias that may affect the cumulative evidence (e.g., publication bias, selective reporting within studies)	6
Additional analyses	18	Describe methods of additional analyses if done, indicating which were prespecified. This may include, but not be limited to, the following: Sensitivity or subgroup	6

		analyses; Meta-regression analyses; Alternative formulations of the treatment network; and Use of alternative prior distributions for Bayesian analyses (if applicable).	
**RESULTS**			
Study selection	19	Give numbers of studies screened, assessed for eligibility, and included in the review, with reasons for exclusions at each stage, ideally with a flow diagram.	6
Presentation of network structure	20	Provide a network graph of the included studies to enable visualization of the geometry of the treatment network.	6
Summary of network geometry	21	Provide a brief overview of characteristics of the treatment network. This may include commentary on the abundance of trials and randomized patients for the different interventions and pairwise comparisons in the network, gaps of evidence in the treatment network, and potential biases reflected by the network structure.	6-7
Study characteristics	22	For each study, present characteristics for which data were extracted (e.g., study size, PICOS, follow-up period) and provide the citations.	6-7
Risk of bias within studies	23	Present data on risk of bias of each study and, if available, any outcome level assessment.	6-7
Results of individual studies	24	For all outcomes considered (benefits or harms), present, for each study: 1) simple summary data for each intervention group, and 2) effect estimates and confidence intervals. Modified approaches may be needed to deal with information from larger networks.	7-8
Synthesis of results	25	Present results of each meta-analysis done, including confidence/credible intervals. In larger networks, authors may focus on comparisons versus a particular comparator (e.g., placebo or standard care), with full findings presented in an appendix. League tables and forest plots may be considered to summarize pairwise comparisons. If additional summary measures were explored (such as treatment rankings), these should also be presented.	7-8
Exploration for inconsistency	26	Describe results from investigations of inconsistency. This may include such information as measures of model fit to compare consistency and inconsistency models, P values from statistical tests, or summary of inconsistency estimates from different parts of the treatment network.	8
Risk of bias across studies	27	Present results of any assessment of risk of bias across studies for the evidence base being studied.	8
Results of additional analyses	28	Give results of additional analyses, if done (e.g., sensitivity or subgroup analyses, meta-regression analyses, alternative network geometries studied, alternative choice of prior distributions for Bayesian analyses, and so forth).	9
**DISCUSSION**			

Summary of evidence	29	Summarize the main findings, including the strength of evidence for each main outcome; consider their relevance to key groups (e.g., health care providers, researchers, and policymakers).	9-10
Limitations	30	Discuss limitations at study and outcome level (e.g., risk of bias), and at review level (e.g., incomplete retrieval of identified research, reporting bias). Comment on the validity of the assumptions, such as transitivity and consistency. Comment on any concerns regarding network geometry (e.g., avoidance of certain comparisons).	10
Conclusions	31	Provide a general interpretation of the results in the context of other evidence, and implications for future research.	10-11
**FUNDING**			
Funding	32	Describe sources of funding for the systematic review and other support (e.g., supply of data); role of funders for the systematic review. This should also include information regarding whether funding has been received from manufacturers of treatments in the network and/or whether some of the authors are content experts with professional conflicts of interest that could affect use of treatments in the network.	11

Search strategy：

S1. Search strategy

Search strategy of Pubmed

#1 Esophageal Neoplasm[MeSH Terms]
#2 Esophagus Neoplasm[Title/Abstract]
#3 Esophagus Neoplasms[Title/Abstract]
#4 Cancer of Esophagus[Title/Abstract]
#5 Cancer of the Esophagus[Title/Abstract]
#6 Esophagus Cancer[Title/Abstract]
#7 Esophagus Cancers[Title/Abstract]
#8 Esophageal Cancer[Title/Abstract]
#9 Esophageal Cancers[Title/Abstract]
#10 Esophageal Carcinoma[Title/Abstract]
#11 #1 OR #2 OR #3 OR #4 OR #5 OR #6 OR #7 OR #8 OR #9 OR #20

#12 Aidi[Title/Abstract]
#13 Chansu[Title/Abstract]
#14 Toad venom[Title/Abstract]
#15 Compound matrine[Title/Abstract]
#16 Fufangkushen[Title/Abstract]
#17 Compound Kushen[Title/Abstract]
#18 Huachansu[Title/Abstract]
#19 Cinobufacini[Title/Abstract]
#20 Kanglaite[Title/Abstract]
#21 Renshenduotang[Title/Abstract]
#22Ginseng polysacchride[Title/Abstract]
#23Tongkening[Title/Abstract]
#24 Wutou[Title/Abstract]
#25 Xiaoaiping[Title/Abstract]
#26 Marsdenia Tenacissima[Title/Abstract]
#27 Yadanziyouru[Title/Abstract]
#28 Javanica oil emulsion[Title/Abstract]
#29 Zhulingduotang[Title/Abstract]
#30 Polyporus[Title/Abstract]
#31 Delisheng[Title/Abstract]
#32 Shenqifuzheng[Title/Abstract]
#33 Yuanqinzhitong[Title/Abstract]
#34 Huangqi duotang[Title/Abstract]
#35 Astragalus polysaccharides[Title/Abstract]
#36 Kangai[Title/Abstract]

#37 Shenfu[Title/Abstract]
#38 Huangqi[Title/Abstract]
#39 Astragalus[Title/Abstract]
#40 Xiangguduotang[Title/Abstract]
#41 Lentinan[Title/Abstract]
#42 Shenmai[Title/Abstract]
#43 Lanxiangxi[Title/Abstract]
#44 Elemene[Title/Abstract]
#45 #12 OR #13 OR #14 OR #15 OR #16 OR #17 OR #18 OR #19 OR #20 OR #21 OR #22 OR #23 OR #24 OR #25 OR #26 OR #27 OR #28 OR #29 OR #30 OR #31 OR #32 OR #33 OR #34 OR #35 OR #36 OR #37 OR #38 OR #39 OR #40 OR #41 OR #42 OR #43 #44

#46 randomized controlled trial[Publication Type]
#47 controlled clinical trial[Publication Type]
#48 randomized[Title/Abstract]
49 placebo[Title/Abstract]
#50 randomly[Title/Abstract]
#51 trial[Title/Abstract]
#52 groups[Title/Abstract]
#53 "drug therapy" [Subheading]
#54 #44 OR #45 OR #46 OR #47 OR #48 OR #49 OR #50 OR #51
#55 animals[MeSH Terms]
#56 humans[MeSH Terms]
#57 #55 NOT #56
#58 #54 NOT #57
#59 #11 AND #45 AND #58

Search strategy of Embase
#1 random*
#2 placebo*
#3 doubl* adj blind*
#4 singl* adj blind*
#5 assign*
#6 allocat*
#7 “double-blind procedure”/exp
#8 ”randomized controlled trial”/exp
#9”single-blind procedure”/exp
#10 #1 or #2 or #3 or #4 or #5 or #6 or #7 or #8 or #9

#11 "Esophageal Neoplasm'/exp
#12 Esophagus Neoplasm

#13 Esophagus Neoplasms
#14 Cancer of Esophagus
#15 Cancer of the Esophagus
#16 Esophagus Cancer
#17 Esophagus Cancers
#18 Esophageal Cancer
#19 Esophageal Cancers
#20 Esophageal Carcinoma
#21 Esophagus Carcinoma
#22 #11 OR #12 OR #13 OR #14 OR #15 OR #16 OR #17 OR #18 OR #19 OR #20 OR #21

#23 aidi
#24 chansu
#25 toad AND venom
#26 compound AND matrine
#27 fufangkushen
#28 huachansu
#29 cinobufacini
#30 kanglaite
#31 renshenduotang
#32 ginseng AND polysacchride
#33 tongkening
#34 wutou
#35 xiaoaiping
#36 marsdenia AND tenacissima
#37 yadanziyouru
#38 javanica AND oil AND emulsion
#39 zhulingduotang
#40 polyporus
#41 delisheng
#42 shenqifuzheng
#43 yuanqinzhitong
#44 huangqi AND duotang
#45 astragalus AND polysaccharides
#46 kangai
#47 shenfu
#48 huangqi
#49 astragalus
#50 xiangguduotang
#51 lentinan
#52 shenmai

#53 lanxiangxi
#54 elemene
#55 #23 OR #24 OR #25 OR #26 OR #27 OR #28 OR #29 OR #30 OR #31 OR #32 OR #33 OR #34 OR #35 OR #36 OR #37 OR #38 OR #39 OR #40 OR #41 OR #42 OR #43 OR #44 OR #45 OR #46 OR #47 OR #48 OR #49 OR #50 OR #51 OR #52 OR #53 OR #54
#56 #10 AND #19 AND #52

PRISMA 2009 Flow Diagram

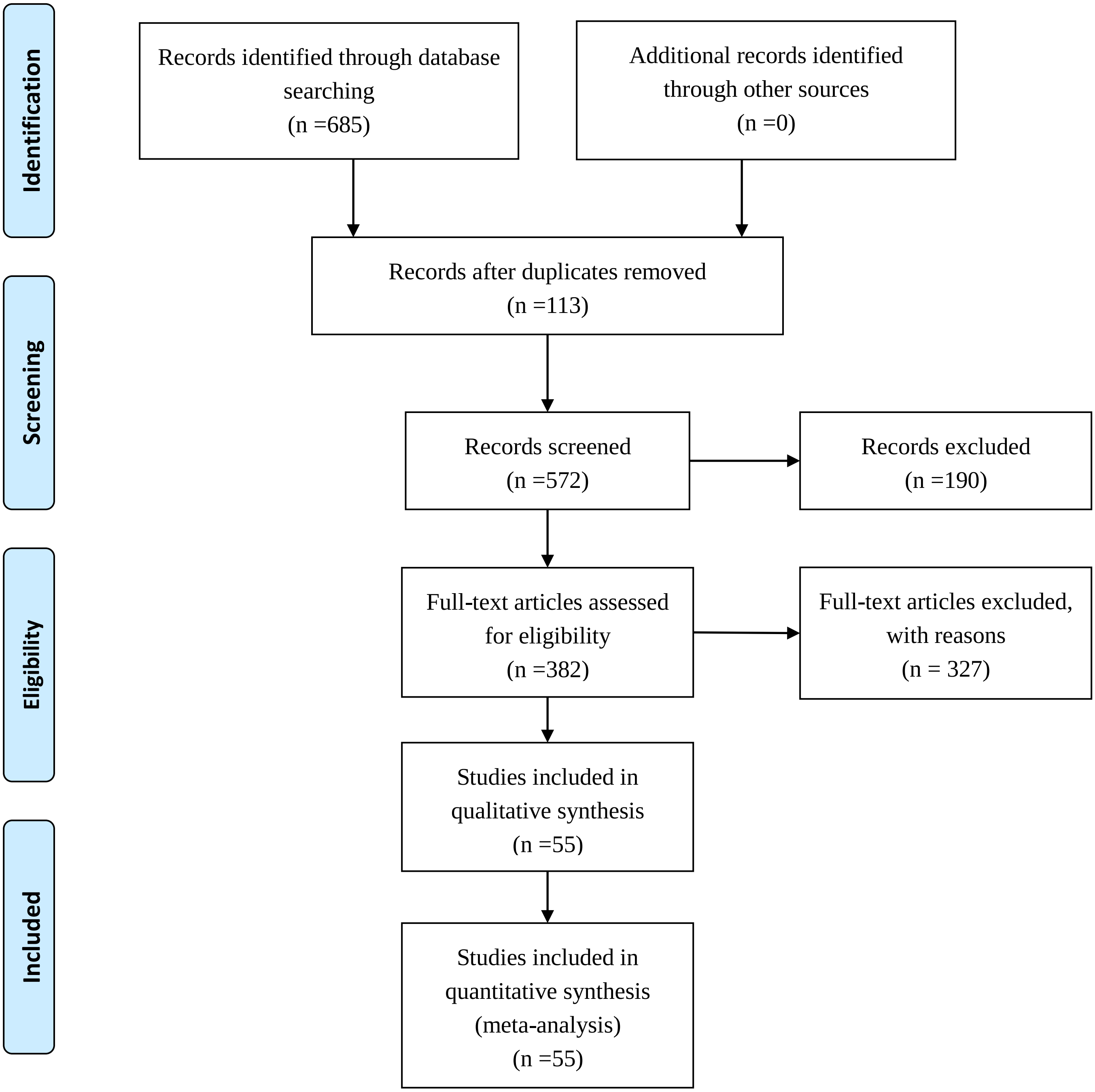

From: Moher D, Liberati A, Tetzlaff J, Altman DG, The PRISMA Group (2009). *P*referred *R*eporting *I*tems for *S*ystematic Reviews and *M*eta-*A*nalyses: The PRISMA Statement. PLoS Med 6(6): e1000097. doi:10.1371/journal.pmed1000097

For more information, visit www.prisma-statement.org.
