# Supplementary figures and images for: The Optimal Chinese Herbal Injections for Use With Radiotherapy to Treat Esophageal Cancer: A Systematic Review and Bayesian Network Meta-Analysis
Source: Front Pharmacol. 2019 Jan 4;9:1470. doi: 10.3389/fphar.2018.01470 (PMC6329258; doi:10.3389/fphar.2018.01470)

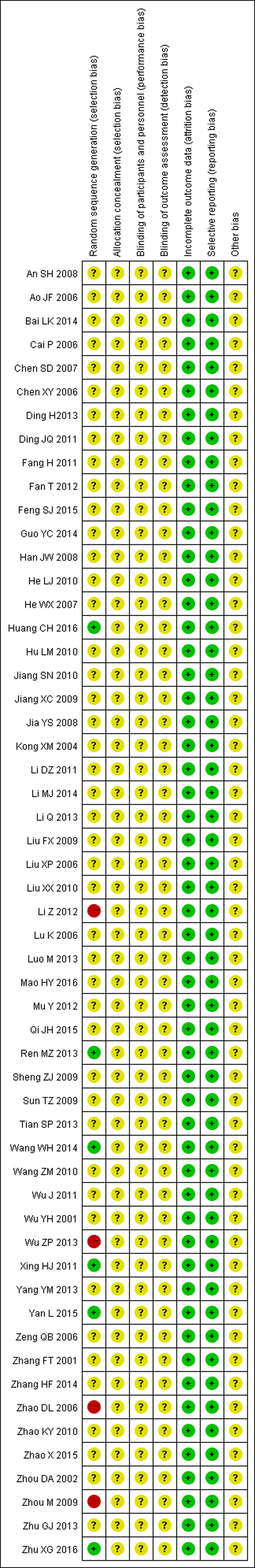

Supplement: Figure S1 — Risk-of-bias summary. [file Image_1.tif]
