# Supplementary material for: The Optimal Chinese Herbal Injections for Use With Radiotherapy to Treat Esophageal Cancer: A Systematic Review and Bayesian Network Meta-Analysis
Source: Front Pharmacol. 2019 Jan 4;9:1470. doi: 10.3389/fphar.2018.01470 (PMC6329258; doi:10.3389/fphar.2018.01470)
Supplement: File S1 — Included RCTs. [file Presentation_1.pdf]

References

1. Yang,Y.M., Song, G.W., 2013. Aidi injection on the quality of life of patients with esophageal cancer clinical research. Med Aesthetics Cosmetology. (11), 27-28.

2. Fang, H., 2011. Aidi injection on quality of life in patients with esophageal cancer clinical research. Health Required. (6), 30.

3. Zeng, Q.B., Yang, X.H., 2006. Application of aidi injection combined with radiotherapy in the treatment of esophageal cancer.Anthology of Med. 25(3), 418-419.

4. Mao, H.Y., Wang, Z., Yuan, X., Han, F., Dong, J.D., 2016. Clinical effect of aidi injection combined with radiotherapy in the treatment of esophageal cancer. J Clin Med Prac. 20(24), 97-99.

5. Zhao, K.Y., Ren, Q.Y., Liu, F.L., Wang, Z.X., Yu, J.M., 2010. Efficacy of aidi injection combined with radiotherapy in the treatment of advanced esophageal cancer.National Med Frontiers China. 05(15), 41,46.

6. Guo, Y.C., Wang, X.G., Chen, S.P., Wen, M.L., Guo, H.S., 2014. Aidi injection combined with three-dimensional conformal radiotherapy in the treatment of advanced esophageal cancer in 50 cases. Shaanxi J Tradit Chi Med. (5), 524-525.

7. Li, M.J., Li, X.Z., Wang, Y.F., Sheng, Y.X., 2014. Effect of aidi injection combined with intensive radiotherapy in the treatment of esophageal cancer. Chin Prim Health Care. 28(9), 108-109.

8. Lu, K., Ke, J., Liu, J.J., Wu, X.J., 2006.Clinical observation radiotherapy combined with aidi-injection in the treatment of esophageal carcinoma.Sichuan J Cancer Control. 19(4), 236-238.

9. Hu, L.T., Liao, T.L., Liu, S.J., 2010.Clinical observation on the treatment of esophageal cancer by aidi injection combined with accelerated posterior hypersection conformal radiotherapy.Nei Mongol J Tradit Chinese Med. 29(16), 91-91.

10. Bai, L.K., 2014.Application of aidi injection in radiotherapy of esophageal cancer in the elderly. China Heal Care &Nutr. 24(5), 2833.

11. Zhao, X. 2015.Clinical report on the curative effect of three dimensional conformal radiotherapy combined with aidi injection in esophageal cancer. For All Heal. (11), 160-161.

12. Han, J.W., 2008. The role of aidi injection in the radiotherapy of esophageal cancer [J].J Med Forum. 29(11), 68-69.

13. Xing, J.H., 2011. Clinical effect of aidi combined with radiotherapy treatment in the elderly with advanced esophageal cancer. Mod Prev Med. 38(19), 4064-4065,4067.

14. Wu, Y.H., 2001. Application of aidi injection in the radiotherapy of esophageal cancer.China J Cancer Prev Treat. 8(6), 663-664.

15. Liu, X.P., Wang, F.,2006. Radiotherapy combined with addie injection for the treatment of advanced esophageal cancer in the elderly. Chinese J Surg IntegTradit West Med. 12(2), 109-110.

16. Jiang, S.N., Yang, F., Ma, Z.M., 2010. Clinical observation of sodium cantharidate combined with three-dimensional conformal radiotherapy in the treatment of esophageal cancer. J Qiqihar Med Coll. 31(20), 3258-3259.

17. Zhou, M., Shi, J.J., Yan, B.C., 2009. Observation on effect of shenmai injection as an adjuvant therapy on un-resectable esophageal carcinoma. Int Med Heal Guid News. 15(11), 35-37.

18. Zhu, G.J., 2013. Clinical report on the curative effect of esophageal cancer combined with accelerated ultrasegmental radiotherapy combined with shenqifuzheng injection. For All Heal. (10), 5.

19. Cai, P., Liu, Q.S., Chen, H.H., Li, Y.X., Li, X.H., 2006.Clinical analysis of esophageal cancer patients treated by combined composite Kushen injection and radiotherapy. Cancer Res Clin. 18(6), 395-396.

20. Yan, L., Ba, N., Wang, L.J., Zheng, X.K., Zhang, J., Zhang, H.Q., Xing, X., 2015.Study on the application of compound radix sophoraeflavescentis in the three dimensional conformal intensity modulated radiotherapy for esophageal carcinoma. Guangming J Chinese Med. (8), 1826-1828.

21. Sun, T.Z., 2009.Compound kushen injection combined with radiotherapy in the treatment of advanced esophageal cancer. China Prac J Med. 36(24), 70-71.

22. Huang, C.H., Tan, C., Cai, J., 2016.Clinical value of compound kushen injection combined with radiotherapy in the treatment of advanced esophageal cancer in the elderly. J Nantong Univ. 36(6), 600-602.

23. Ao, J.F., 2006. Observation on the short-term curative effect of compound kushen injection combined with radiotherapy in the treatment of middle and late esophageal cancer. PracPrev Med. 13(3), 743-743.

24. Chen, X.Y., Dai, X.B., Zhang, L., Zhang, X.P., Zhang, J., Zhu, Y.X., Wu, Y.Z., Zhao, B., 2006.Clinical study on combined radiotherapy of compound kushen injection in the treatment of esophageal cancer. Pharmaceut Care Res. 6(1),45-47.

25. Sheng, Z.J., Sun, J., Feng, L.J., 2009. Clinical efficacy of compound knshen injection combined with three dimensional conformal radiation therapy for senile patients with stage Ⅲ esophageal carcinoma.Eval and Anal Drug-Use in Hosp China. 9(12), 936-937.

26. Ding, J.Q., Li, J.Z., Li, R.Q., 2011. Clinical observation of compound kushen injection combined with esophageal cancer in the later stage of accelerated ultrasegmented radiotherapy. China Pharm. (20), 1870-1872.

27. Li, Z., 2012. Clinical effect of compound kushen injection on esophageal cancer radiotherapy was observed. J Mil Surgeonin in Southwest China. 14(1), 152-153.

28. Luo, M., Zhu, Y.Y., 2013. Effect of compound kushen injection on radiation esophagitis. Med Front. (15), 179-179.

29. Liu, F.X., 2009. Clinical observation of yansu injection combined with radiotherapy in the treatment of esophageal cancer. China Prac Med. 4(29), 131-132.

30. Zhou, D.A., Wang, J., Qiao, X.Y., 2002. Healing effects analysis of radiotherapy concomitant with Huachansu on esophageal cancer. Mod J IntegTradit Chinese and Western Med. 11(2), 101-102.

31. Tian, S.P., Shi, S.X., Yang, B.W., 2013. Clinical observation on treatment of senile esophageal cancer with bufalin combined with radiotherapy. Chinese J Mod Drug Appl. 7(16), 126.

32. Zhang, F.T., Li, N., 2001.Analysis of efficacy of bufalin combined with radiotherapy in the treatment of esophageal cancer.Mod J IntegTradit Chinese and Western Med. 10(16), 1553-1574.

33. Wang, Z.M., Li, H.B., Li, Y.C., Zhang, X.Y., 2010.Stereotactic radiotherapy combined with cinobufotoxin for treating advanced esophagus carcinoma in 28 cases. China Pharmaceuti. 19(17), 61-62.

34. He, W.X., Zhu, Y.Y., Li, J., 2007. Effect of astragalus polysaccharide for injection on quality of life of patients with esophageal cancer radiotherapy.Jiangxi J Tradit Chinese Med. 38(2), 37-38.

35. Fan, T., 2012.Clinical observation of astragalus injection combined with radiotherapy in the treatment of esophageal cancer. Nati Med Front China. (20), 42.

36. An, S.H., 2008. The effect of kangai injection on the radiotherapy of esophageal cancer in the elderly. Proceedings of the second Chinese and Japanese academic conference on interventional therapy of cancer.Chinese medical association oncology branch, Chinese cancer society. 1.

37. Zhao, D.L., Zhang, X.Z., Du, P.R., 2006.Clinical study on quality of life for radiotherapy combined with kangai solution.Chinese J Cancer Prev Treat. 13(2), 146-147.

38. Wang, W.H., Chen, Q., Jiang, D.W., 2014. Clinical study of Kangai Injection combined with radiotherapy in treatment of advanced esophageal carcinoma.Drugs & Clinic. (9), 1032-1035.

39. Mu, Y., Yuan, Y.M., Zhou, J., 2012. Clinical observation of 50 cases of advanced esophageal cancer treated by kangai injection combined with radiation. Guiding J Tradit Chinese Med Pharmacol. 18(11), 35-37.

40. Zhang, H.F., 2014. Observation of kang'ai injection combined with radiotherapy for advanced esophageal carcinoma: a clinical comparative study. Guide of China Med. (22), 29-30.

41. Wu, Z.P., Wu, X.R., Ji, W., Ji, F., 2013. Observation on curative effect of kangai injection combined with three-dimensional conformal radiotherapy in the treatment of elderly esophageal cancer. Zhejiang J IntegTradit Chinese and Western Med. (4), 274-275.

42. Ren, M.Z., Gao, G.W., Wang, Z.H., 2013. Clinical analysis of 28 cases of senile esophageal cancer treated by three-dimensional conformal radiotherapy combined with kangai injection. Shanxi Med J. (18), 1039-1041.

43. Ding, H., Liu, X.Y., Wu, Z.J., Liu, J.B., Guan, Z.F., 2013.The radio sensitizing effect of elemene injection in the treatment of advanced esophageal cancer.J Nantong Univ. 33(3), 182-184.

44. Zhu, X.G., Li J.L., Chen, B.P., Gao, Y., Zhu, X.L., Chen, Y.L., 2016. Effect of elemene on the therapeutic effect of esophageal cancer in three-dimensional conformal radiotherapy. China Pharm. (14), 1963-1966.

45. Wu, J., Ye, J.K., 2011. Clinical observation on esophageal carcinoma treated by radiotherapy combined with lentinan injection.J Med Res. 40(4), 128-130.

46. Feng, S.J., 2015. Clinical study on the therapeutic effect of injection of crow's gall cream combined with radiotherapy in patients with esophageal cancer. China Heal Care Nutr. (12), 130-131.

47. Li, D.Z., Li, G.M., Wen, S.M., 2011. 28 cases of recurrent esophageal cancer after radiotherapy were treated with three-dimensional conformal radiotherapy combined with crow's gall oil emulsion injection. Chongqing Med. 40(2), 170-171.

47. Li, Q., Deng, S.H., Chen, P., 2013. Observation on the curative effect of enhanced radiotherapy combined with crow's gall oil emulsion in the treatment of recurrent esophageal cancer. Shanxi Med J. 42(2), 86-87.

48. Jia, Y.S., Wu, S.Q., Lv, S.L., 2008. Clinical analysis on the treatment of advanced esophageal cancer by corvidae oil emulsion and radiation. China J Chinese Mater Med. 33(17), 2174-2176.

49. Chen, S.D., Wang, D.F., Jiang, Q.J., Zhu, G.M., 2007.Clinical observation on curative effect of crow gall - seed oil - emulsion injection in advanced esophageal cancer radiotherapy. J PracTradit Chinese Internal Med. 21(9), 78-79.

50. Jiang, X.C., Huo, S.X., 2009. Clinical study of yadanzi oil emulsion injection improving efficacy on radiotherapy in treatment of esophageal cancer.The Chinese and foreign health abstract. 6(21), 35-36.

51. Kong, X.M., Xu, L.J., Zhang, N., Liu, S.Y., 2004.Clinical observation on treating advanced esophageal cancer with emulsion injection of seminal pil plus radiotherapy. Zhejiang J integrated Tradit Chinese and Western Med. 14(6), 339-340.

52. Liu, X.X., Zhu, H., 2010. 28 cases of esophageal cancer were treated by crow's gall milk injection combined with radiotherapy. Jiangxi J Tradit Chinese Med. 41(3), 42-43.

53. He, L.J., Luo, H.Q., Xiang, L., 2010. Efficacy of bruceajavanica oil emulsion combined radiotherapy on treating advanced esophageal carcionma. Chinese J Exp Tradit Med Formulae. 16(7), 212-214.

54. Qi, J.H., Zhang, L.Z., 2015. Clinical observation on curative effect of crow gallbladder oil emulsion injection combined with conformal radiotherapy on local advanced esophageal carcinoma. Henan Med Res. (2), 60-62.
