# Supplementary material for: The Optimal Chinese Herbal Injections for Use With Radiotherapy to Treat Esophageal Cancer: A Systematic Review and Bayesian Network Meta-Analysis
Source: Front Pharmacol. 2019 Jan 4;9:1470. doi: 10.3389/fphar.2018.01470 (PMC6329258; doi:10.3389/fphar.2018.01470)
Supplement: File S2 — Quality evaluation of the included RCTs. [file Presentation_2.pdf]

S2. Quality Evaluation of the Included RCTs

Study ID	Randomized method	Follow-up	Blind method	Allocation concealment	Reasons of withdrawal	Inclusion and exclusion criteria	ADRs	Statistical methods	Foundations	Medical ethics	Scores
Yang YM 2013	0	0	0	0	0	1	0	1	0	1	3
Fang H 2011	0	0	0	0	0	1	0	1	0	1	3
Zeng QB 2006	0	0	0	0	0	1	1	0	0	0	2
Mao HY 2016	0	0	0	0	0	1	1	0	0	0	2
Zhao KY 2010	0	0	0	0	0	1	1	1	0	0	3
Guo YC 2014	0	0	0	0	0	1	1	1	0	0	3
Li MJ 2014	0	0	0	0	0	1	1	1	0	1	4
Lu K 2006	0	0	0	0	0	1	1	1	0	0	3
Hu LM 2010	0	1	0	0	0	1	1	1	0	0	4
Bai LK 2014	0	0	0	0	0	1	1	1	0	0	3
Zhao X 2015	0	1	0	0	0	1	1	0	0	0	3
Han JW 2008	0	0	0	0	0	1	1	0	0	0	2
Xing HJ 2011	1	1	0	0	0	1	1	1	0	1	6
Wu YH 2001	0	1	0	0	0	1	1	0	0	0	3
Liu XP 2006	0	0	0	0	0	1	1	1	0	0	3
Jiang SN 2010	0	0	0	0	0	1	0	0	0	0	1
Zhou M 2009	1	1	0	0	0	1	1	1	0	0	5
Zhu GJ 2013	0	1	0	0	0	1	1	0	0	0	3
Cai P 2006	0	1	0	0	0	1	1	1	0	0	4
Yan L 2015	1	0	0	0	0	1	1	1	0	1	5

Sun TZ 2009	0	1	0	0	0	1	1	0	0	0	3
Huang CH 2016	1	1	0	0	0	1	1	1	0	0	5
Ao JF 2006	0	0	0	0	0	1	1	1	0	0	3
Chen XY 2006	0	1	0	0	0	1	1	1	0	0	4
Sheng ZJ 2009	0	0	0	0	0	1	1	1	0	0	3
Ding JQ 2011	0	1	0	0	0	1	1	1	0	0	4
Li Z 2012	1	0	0	0	0	1	1	1	0	0	4
Luo M 2013	0	0	0	0	0	1	1	0	0	0	2
Liu FX 2009	0	0	0	0	0	1	1	0	0	0	2
Zhou DA 2002	0	0	0	0	0	1	1	1	0	0	3
Tian SP 2013	0	0	0	0	0	1	1	0	0	0	2
Zhang FT 2001	0	1	0	0	0	1	0	0	0	0	2
Wang ZM 2010	0	0	0	0	0	1	1	1	0	0	3
He WX 2007	0	0	0	0	0	1	0	1	0	1	3
Fan T 2012	0	0	0	0	0	1	1	0	0	0	2
An SH 2008	0	0	0	0	0	1	0	0	0	0	1
Zhao DL 2006	1	0	0	0	0	1	0	1	0	0	3
Wang WH 2014	1	0	0	0	0	1	1	1	0	1	5
Mu Y 2012	0	0	0	0	0	1	0	1	0	0	2
Zhang HF 2014	0	0	0	0	0	1	1	1	0	0	3
Wu ZP 2013	1	1	0	0	0	1	1	1	0	0	5
Ren MZ 2013	1	0	0	0	0	1	1	1	0	0	4
Ding H2013	0	0	0	0	0	1	1	1	1	0	4

Zhu XG 2016	1	1	0	0	0	1	1	1	0	1	6
Wu J 2011	0	0	0	0	0	1	1	1	0	1	4
Feng SJ 2015	0	0	0	0	0	1	1	1	0	0	3
Li DZ 2011	0	0	0	0	0	1	1	1	0	0	3
Li Q 2013	0	1	0	0	0	1	1	1	0	0	4
Jia YS 2008	0	1	0	0	0	1	1	1	0	0	4
Chen SD 2007	0	0	0	0	0	1	1	1	0	0	3
Jiang XC 2009	0	0	0	0	0	1	1	1	0	0	3
Kong XM 2004	0	1	0	0	0	1	1	0	0	0	3
Liu XX 2010	0	0	0	0	0	1	1	1	0	0	3
He LJ 2010	0	0	0	0	0	1	1	1	0	0	3
Qi JH 2015	0	0	0	0	0	1	1	0	0	0	2

Note:1=report; 0=not report
